# Magneto-Optical Trap Field Characterization with the Directional Hanle Effect

**DOI:** 10.1038/s41598-019-45324-7

**Published:** 2019-06-20

**Authors:** Jarom S. Jackson, Dallin S. Durfee

**Affiliations:** Department of Physics and Astronomy, Brigham Young University, Provo, Utah 84602 USA

**Keywords:** Atomic and molecular physics, Atomic and molecular interactions with photons

## Abstract

We demonstrate the use of spatial emission patterns to measure magnetic fields. The directional aspect of the Hanle effect gives a direct, visual presentation of the magnetic fields, in which brighter fluorescence indicates larger fields. It can be used to determine the direction as well as the magnitude of the field. It is particularly well suited for characterizing and aligning magneto-optical traps, requiring little or no additional equipment or setup beyond what is ordinarily used in a magneto-optical trap, and being most sensitive to fields of the size typically present in a magneto-optical trap.

## Introduction

In 1924, Wilhelm Hanle presented a theory explaining the depolarization of light scattered by an atomic vapor in a magnetic field. This effect, known as the Hanle effect, has been used to determine atomic state lifetimes^1^ and to measure magnetic fields^2–4^ and phenomena related to magnetic fields^5,6^. Other manifestations associated with the Hanle effect include level-crossing spectroscopy^7,8^, and a “mechanical” Hanle effect^9^. Unlike Hall probe measurements, field measurements using the Hanle effect require no sensor, just the atoms themselves, in the region being measured. Also, unlike many spectroscopic methods, first-order Doppler shifts do not degrade measurement precision^8^.

While most Hanle effect studies focus on depolarization, the interaction of atoms with magnetic fields *also* affects the *spatial pattern* of the scattered light^8,10–12^. We refer to the field-dependent spatial radiation pattern as the “directional” Hanle effect. Unlike polarization measurements, directional Hanle effect measurements are sensitive to atom density and driving light intensity. But they are not susceptible to errors due to polarization-altering properties of the medium between the measurement region and the detector, and they do not require polarizing elements in the detector or require polarizers to be rotated during the measurement. Adding a polarizer can, however, reveal additional information.

Applications of the directional Hanle effect are possible in astrophysics, laboratory plasmas, and other disciplines where Hanle effect-related techniques have been developed^13–15^ or proposed^16–18^. In this paper we demonstrate the utility of the directional Hanle effect to characterize the magnetic fields used in a magneto-optical trap (MOT). Other fluorescence-based field measurements have been demonstrated^19,20^, some of which have higher precision and/or accuracy, or the ability to measure fields which are smaller or larger than can be done with the directional Hanle effect. But a significant advantage of the directional Hanle effect for MOT alignment and characterization is that it produces an immediate, visual display of the field strength, where brighter fluorescence corresponds to larger fields. Simply looking at the fluorescing atoms, one can see where the field is large or small, and where field zeros are located. One can quickly identify the MOT trapping region as a dark spot in the fluorescence. This visual feedback is particularly useful when aligning laser beams for a MOT. Furthermore, directional Hanle effect measurements can be made *in*-*situ* without breaking vacuum, using the same atoms used in the MOT.

Another significant advantage of using this method to characterized and align MOTs is that it can be applied with little or no additional equipment beyond what is already present in a typical MOT — usually requiring very little change to the MOT apparatus. Furthermore, it is most sensitive to fields of the magnitude typically present in MOTs, and at which polarization gradient cooling is degraded^21–24^, making it useful for both MOT characterization and for field nulling for sub-Doppler cooling. Because the effect saturates at about the same field magnitude which defines the edge of the trapping region, the size of the dark spot in the fluorescence gives an estimate of the size of the MOT trapping volume. In addition to magnitude, the technique can also be used to determine the field’s direction at every point in the measurement region.

## Description of the Hanle effect

The Hanle effect can be understood qualitatively by treating atoms as classical oscillating electric dipoles. In this picture, similar to what was first proposed by Hanle^4,8^, light induces an oscillating electric dipole moment in an atom. The dipole is initially excited in the direction of the driving light polarization. If a magnetic field is present, the dipole oscillation axis will rotate around the field, changing the polarization of the emitted light. Averaged over time, this reduces the scattered light polarization.

**Figure 1:**
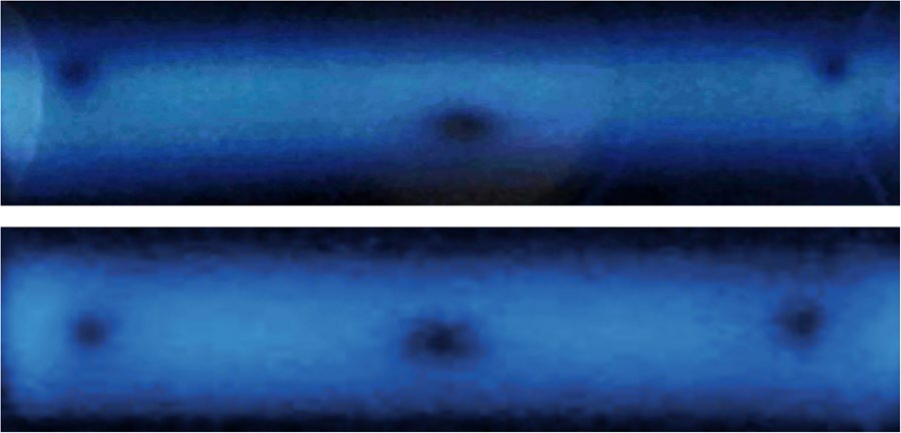
Two pictures of fluorescence from strontium atoms in an inhomogeneous magnetic field. An expanding thermal strontium beam was illuminated with a thin sheet of resonant laser light. With the laser polarization set along the line from the atoms to the camera, zero crossings in the field are clearly visible as dark spots in the scattered light. The width of the spots is related to the local field gradients. The central dark region is the location of the MOT’s trapping volume. The other two dark spots are points at which the laser light sheet intersects with a ring-shaped zero crossing caused by the non-traditional field geometry in our apparatus. The fact that the zero crossings are not collinear in the upper figure revealed that the magnets generating the field had significantly different magnetizations. The lower image was taken after the defective magnet was replaced.

In the *directional* Hanle effect, we consider how the field changes the *spatial radiation pattern* of the scattered light. With no field present, the oscillating dipole emits with a sine-squared dipole emission pattern, and a detector displaced from the atoms in the driving light polarization direction will measure no scattered light. If a magnetic field is present, however, the emission pattern rotates. The strength of the field determines the rotation rate, and the rotation rate, compared to the decay time, determines the average light intensity scattered in the direction of the pump light polarization. An example of this can be seen in Fig. 1, which shows fluorescence from atoms in a region with 3 field zero points^25^. Note that the directional Hanle effect provides a picture of the field strength, viewable with a camera or directly viewable by eye.

## Measurement of MOT fields

**Figure 2:**
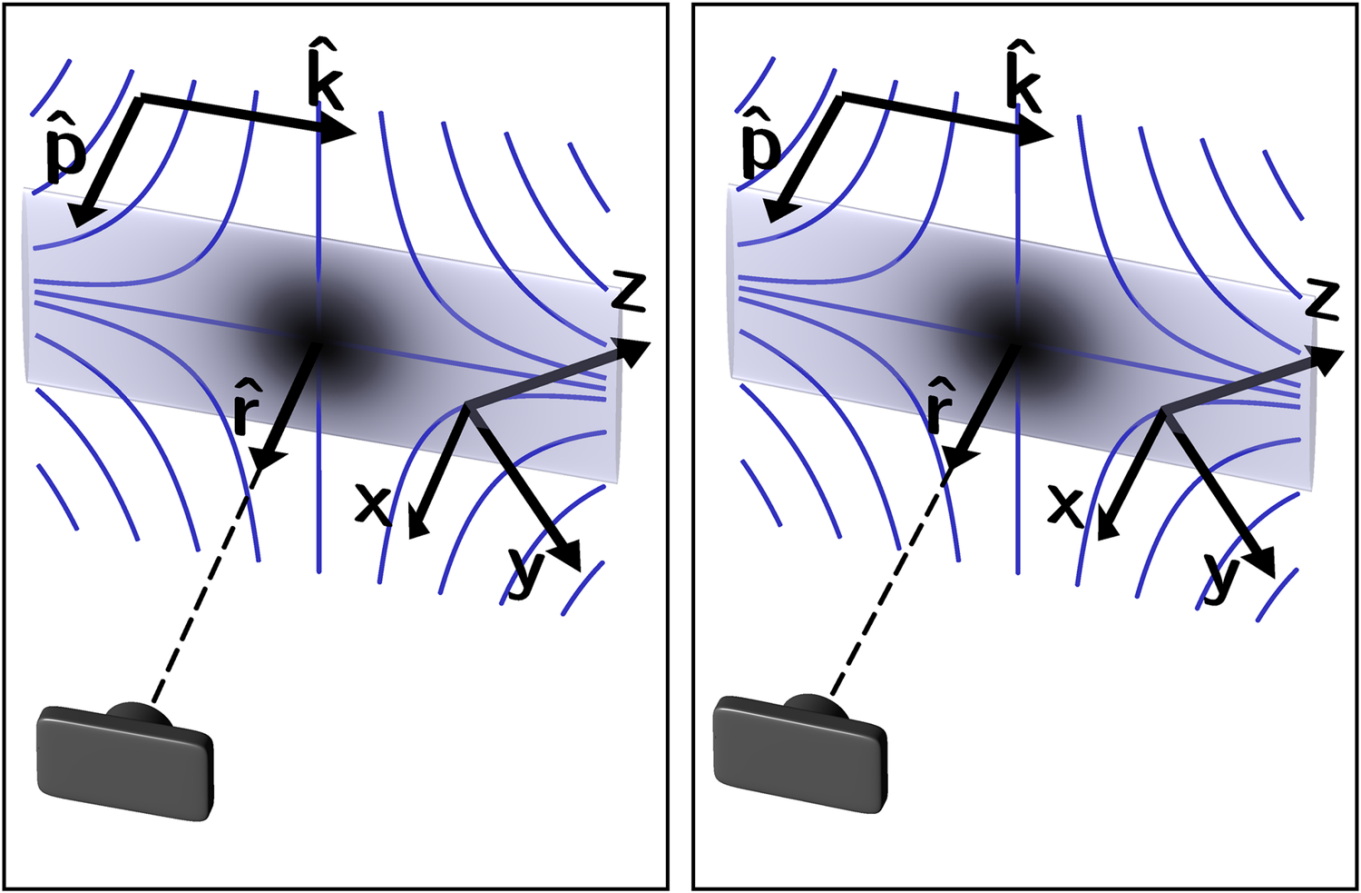
Stereo pair illustration of the coordinate system. The two images are from slightly different perspectives, producing a 3D stereoscopic pair for divergent ‘wall-eyed’ viewing. The sheet of light, polarized in the $\hat{{\bf{p}}}$ direction, is depicted by a rectangle. The dark area in the center of the rectangle depicts a low-field region from which little light scatters toward the camera, shown in the lower left-hand corner displaced from the center of the rectangle in the $\hat{{\bf{r}}}$ direction. The curves represent magnetic field lines. The field direction defines the local *z* axis, and the *x* direction is defined to be normal to the light sheet, which is orthogonal to the laser propagation direction $\hat{{\bf{k}}}$. For most of the discussion in this paper, $\hat{{\bf{x}}}$, $\hat{{\bf{r}}}$, and $\hat{{\bf{p}}}$ are parallel. In the “Field direction” section, however, the utility of taking data with either $\hat{{\bf{p}}}$ or $\hat{{\bf{r}}}$ rotated away from $\hat{{\bf{x}}}$ is discussed.

We used the directional Hanle effect to characterize the inhomogeneous magnetic field in the center of a strontium MOT apparatus^26^. To do this, we blocked all but one laser beam going through the vacuum chamber. With just one beam, there are no cooled, trapped atoms. Instead, thermal atoms scatter light from the remaining beam. A narrow slit was placed in the beam to create a sheet of light passing through the center of the MOT trapping region. The light sheet was aligned to pass through the field zero in the center of the region by adjusting its position to make the central dark spot in Fig. 1 as large and dark as possible. The light scattered by the atoms was measured with a camera displaced from the atoms in a direction normal to the surface of the light sheet, as shown in Fig. 2. This figure also shows the direction of the various coordinates used in our analysis. For most of the measurements described, the driving light sheet was polarized normal to the surface of the light sheet.

In this paper we specifically consider the 5s^2 1^S_0_ to 5s5p ^1^P_1_ resonance transition in strontium. This transition results in a Hanle effect which is well described by the classical model first proposed by Hanle^4,8^. It has a simple structure, such that we do not have to consider effects like the ground-state Hanle effect^27,28^, ground-state coherences and dark resonances^29^, and optical pumping^8^. A more general treatment of the directional Hanle effect is beyond the scope of this paper. However, the Hanle effect is a general phenomena, which has been demonstrated with many atoms, such that this technique should be widely applicable. The master equation derivation presented in the Supplementary Material demonstrates how previous work related to the polarization aspect of the Hanle effect can be extended for use in directional Hanle effect measurements. In systems where optical pumping occurs, the same repump light used to realize the MOT can be employed when taking directional Hanle effect measurements. Because the Hanle effect is a single-scattering-event phenomena, the need to use repumping light should not significantly change how we think about the effect.

To analyze the data in this paper, we used a fully quantum model derived using a master equation, based on a derivation by Avan and Cohen-Tannoudji^11^, but extended to allow for a more general polarization of the driving light. Details of this derivation can be found in the Supplementary Information. For quantitative measurements, weak driving light, such that the peak saturation parameter is much less than one, is preferred. Otherwise the pump light intensity at each point in the image needs to be known with some precision. We assume that the probe field is not large enough for inelastic scattering, and we assume that the atomic vapor is dilute, such that re-scattering of scattered light is negligible. Because of the known symmetry of the field, we assume that the field at every point in the light sheet lies within the plane of the sheet.

For a $j=0$ to $j=1$ transition driven with linearly polarized, narrowband radiation, in the limit of weak pump light, and assuming that the magnetic field is orthogonal to $\hat{{\bf{p}}}$, the intensity scattered in the $\hat{{\bf{p}}}$ direction is proportional to

1 $${I}_{\hat{{\bf{p}}}}=\frac{{\omega }_{{\rm{L}}}^{2}}{{\rm{\Delta }}{\omega }^{4}+2{\rm{\Delta }}{\omega }^{2}({(\frac{{\rm{\Gamma }}}{2})}^{2}-{\omega }_{{\rm{L}}}^{2})+{({(\frac{{\rm{\Gamma }}}{2})}^{2}+{\omega }_{{\rm{L}}}^{2})}^{2}}$$

where Δ$\omega$ is the detuning from resonance (including both laser detuning and Doppler shift), ${\rm{\Gamma }}$ is the natural linewidth of the transition, and the Larmor frequency ${\omega }_{{\rm{L}}}=g{\mu }_{{\rm{B}}}B/\hslash$, where $\hslash$ is Planck’s constant over 2*π*, *μ*_B_ is the Bohr magneton, *B* is the field magnitude, and *g* is the Landé g-factor ($g=1$ for the 5s5p ^1^P_1_ state in strontium). For comparison, the light that would be scattered in a direction *orthogonal* to $\hat{{\bf{p}}}$, in the *absence* of a magnetic field is proportional to

2 $${I}_{{\rm{perp}}}=\frac{1}{{\rm{\Delta }}{\omega }^{2}+{({\rm{\Gamma }}/2)}^{2}}$$

with the same constant of proportionality.

## A simplified model

In addition to the full quantum model we used in our quantitative data analysis and the classical oscillating dipole model used above to qualitatively explain the Hanle effect, an extremely simplified semi-classical model of light scattering can be useful to develop intuition. While this model is not as accurate as the full quantum model, it is easy to visualize the derivation, and it results in a simple equation from which intuition for the directional Hanle effect can be readily gained. The model is somewhat easier to derive than a fully classical model, and it gives additional, complementary understanding of the directional Hanle effect. Further insight can be gained by comparing the results of this model with the full quantum model used in our quantitative analysis.

In the simplified model we treat radiation fields classically, but quantize atomic energy levels. To simplify the results of this model, and to make it easier to gain intuition from the resulting equation, we consider an atom which is excited by an impulse, rather than a continuously driven atom. After absorbing a photon, an atom’s initial state will be a superposition of upper-state magnetic sublevels. Each sublevel emits a classical field, and these fields interfere. Whether they interfere constructively or destructively depends on the relative phase of the emitting states. In this picture, the Hanle effect can be thought of as the averaging of quantum beats in the interference^4,8^.

As with our more complete analysis, we will assume a $j=0$ to $j=1$ transition. This simple level structure makes the mathematics less complicated and more intuitive. For this level structure, our simplified model is in qualitative agreement with the classical oscillating dipole description given above. Furthermore, this is the structure of the transition used in our experiments. Because the field in our measurement region lies within the plane of the light sheet, orthogonal to $\hat{{\bf{p}}}$, the $m=0$ upper state is not excited and can be ignored.

In this simplified model, we will assume Zeeman shifts only affect the relative phase evolution of the upper states, not the overall scattering rate, and further assume that the $m=-\,1$ and the $m=+\,1$ states are populated equally, ignoring effects of laser detuning and Doppler shifts, which would result in different upper-state populations. For broadband driving light, this is approximately true. With narrowband excitation, when the Doppler width of the atoms is much larger than both the natural linewidth and the Zeeman shifts, errors due to this approximation largely wash out when emissions are averaged over the velocity distribution of the atoms. Even when it is not a good approximation, it allows us to find a simple analytical expression to aid intuition.

For the simplified model we assume that each of the two excited states emits the field of a classical rotating dipole (rotating in opposite directions), which decays according to the transition linewidth ${\rm{\Gamma }}$. Due to Zeeman shifts, the components of the field from the two sublevels oscillate at slightly different rates, ${\omega }_{\pm }={\omega }_{0}\pm {\rm{\Delta }}\omega$, where ${\omega }_{0}$ is the unshifted frequency, and ${\rm{\Delta }}\omega ={\mu }_{{\rm{B}}}gB/\hslash$. This causes the relative phases of the two field contributions to drift, altering the radiation field in a way analogous to the field of a classical oscillating dipole with a drifting oscillation axis.

In the far field, the resulting time-averaged intensity emitted in the $\hat{{\bf{p}}}$ direction is an inverted Lorentzian:

3 $$\langle {I}_{{\rm{ensemble}}}\rangle =\frac{{I}_{{\rm{perp}}}}{2}[1-\frac{1}{1+\frac{4{\mu }_{{\rm{B}}}^{2}{g}^{2}}{{\hslash }^{2}{{\rm{\Gamma }}}^{2}}{B}^{2}}],$$

where *I*_perp_ depends on the driving light intensity and the density of atoms, and is equal to the intensity which would be detected with no field if the detector were displaced in a direction perpendicular to $\hat{{\bf{p}}}$. More details related to this simplified, intuitive semi-classical model are contained in the Supplementary Information.

Equation 3 indicates, as expected, that in the limit of zero magnetic field, no light is emitted in the $\hat{{\bf{p}}}$ direction. As the field increases, the intensity grows, approaching an asymptotic limit of *I*_perp_/2. The half-width, half-maximum width of the Lorentzian is

4 $${B}_{{\rm{HWHM}}}=\hslash {\rm{\Gamma }}/2g{\mu }_{{\rm{B}}}.$$

This is the field at which the Larmor frequency is half the natural linewidth. Since the slope of Eq. 3 flattens once *B* is several times *B*_HWHM_, and because optical trapping is inefficient when the Zeeman shift becomes comparable to or larger than the light detuning, which is typically on the order of the natural linewidth in a MOT, the size of the central dark region in the fluorescence is related to the size of the MOT trapping volume.

Because of the shallow slope of Eq. 3 near zero field and the asymptotic saturation at high fields, measurements using the directional Hanle effect are most precise for field magnitudes of order *B*_HWHM_. This is precisely the scale of the fields present in a typical MOT’s trapping region^30^. Additionally, this method is well suited to zeroing fields for the field-free polarization gradient cooling step used in some cold atom experiments^21,24^, as polarization gradient cooling is detrimentally affected by fields of order *B*_HWHM_^22,23^, where this method is most sensitive.

## Theoretical curves

**Figure 3:**
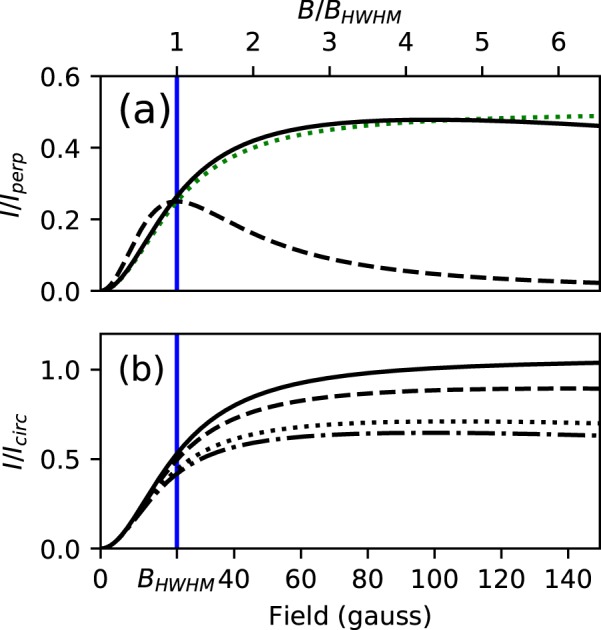
Intensity emitted along the pump light polarization axis vs. field strength. All curves assume that the field direction $\hat{{\bf{z}}}$ is in the plane of the light sheet. In addition to the field in gauss, the *x* axis is also labeled using the unitless quantity *B*/*B*_HWHM_ to make it easier to approximate what the curves would be like for other atomic transitions. In (**a**) the theoretically expected light intensity scattered in the $\hat{{\bf{p}}}$ direction, normalized to *I*_perp_, is plotted as a function of magnetic field. The full quantum model, applied to motionless atoms with the driving light tuned to resonance, is shown with a dashed line. The solid line represents the full model applied to a thermal beam of atoms, as described in the text. The dotted line is the simplified model. In (**b**) the full model, averaged over the thermal distribution of the atoms, is shown normalized to the average of *I*_LH_ and *I*_RH_ (discussed in the section on calibration). The solid/dashed/dotted/dash-dotted lines represent magnetic fields at an angle of 0/30/60/90 degrees from the $\hat{{\bf{k}}}$ direction. In both (**a**,**b**) the blue vertical line represents *B*_HWHM_.

The expression in Eq. 3 is graphed in Fig. 3(a). Also shown are curves generated using the full model of Eq. 1 which we used to analyze data. In the simplified model, laser intensity, detuning, and Doppler shifts only impact *I*_perp_, not the overall curve shape. In the full model, these factors are more important. We assumed a thermal strontium beam from an oven with a temperature of 509 °C propagating in the opposite direction from the driving light, which was tuned to cancel the Doppler shift at the peak of the velocity distribution. For the full model curves, at each field magnitude, ${I}_{\hat{{\bf{p}}}}$ and *I*_perp_ were first averaged over the atomic thermal distribution. Then the two averages were divided to generate the curves in Fig. 3(a).

Figure 3(a) shows that for a gas of thermal atoms with Zeeman shifts that are small compared to the Doppler broadening, the simplified solution is a good representation of the effect, deviating from the full model by only a few percent. While many treatments of the Hanle effect using broadband excitation result in similar Lorentzian curves^1^, this figure illustrates that Lorentzian curves are also useful under narrowband excitation as long as the Doppler broadening is large. The errors introduced into our simplified model by assuming an impulse drive and constant scattering rates mostly vanish when both models are normalized to *I*_perp_, as the semi-classical calculation of *I*_perp_ includes those same assumptions.

For hot atoms, the scattered intensity in the full model initially increases somewhat more rapidly with field than the simplified model. Atoms whose Doppler shift makes them not exactly resonant with the pump light will have one upper state Zeeman shifted *closer* and the other *further* from resonance, such that one state scatters more than the other. This makes complete destructive interference impossible. At high fields, such that the magnitude of the Zeeman shift surpasses both the natural and the Doppler linewidth, the bulk of the atoms in the distribution get shifted from resonance, and the intensity in the full model asymptotically approaches zero with increasing field. This effect is not accounted for in the simplified, semi-classical model. While readily visible in Fig. 3(a) for cold atoms, for the thermal distribution of atoms in our experiment this effect only becomes significant at fields higher than those shown in Fig. 3.

## Calibration

To make quantitative field measurements, both the simplified and complete quantum model require some knowledge related to the light intensity and atomic density at each location in the image. In the limit of low drive intensity (such that the saturation parameter $s\ll 1$), only the product of the two must be known. In this limit, the intensity *I*_perp_ is proportional to this product. So a simple way to calibrate field measurements, without needing to know the atom density or light intensity at any point in the measurement region, is to zero the magnetic field, rotate the driving light polarization such that it is orthogonal to $\hat{{\bf{r}}}$, and directly measure *I*_perp_. In our setup, we were unable to easily remove the magnets, and were not able to make this measurement.

Another approach is to make assumptions about the spatial distribution of the driving light and the atomic vapor, and about the field at certain locations. For example, we achieved results consistent with the expected field by assuming a constant vapor density and assuming that the light scattered along vertical slices half-way between the zeros in Fig. 1 was near the maximum of the curve in Fig. 3(a). While the field is not constant along these slices, with these assumptions, and assuming a collimated laser beam, we obtained results which fit the expected shape of the field quite well. However, the overall magnitude of the field and the size of the field gradients at the location of the central zero which were determined with this method were very dependent on where on the curve of Fig. 3(a) we assumed the vertical slices to be.

A better approach, when the field cannot be zeroed, is to measure the light scattered by circularly polarized driving fields. The amount of light scattered in the $\hat{{\bf{r}}}$ direction is less sensitive to the field when circularly polarized pump light is used (see Fig. 6(f)). Simply dividing by an image made with circularly polarized light and using the simplified model yields a reasonable result. To obtain more accurate results, we extended Avan and Cohen-Tannoudji’s full quantum model of the Hanle effect^11^ to allow circularly polarized driving light. In the limit of low pump intensity, and assuming the magnetic field lies within the plane of the light sheet, the light scattered to the camera by a circularly polarized pump beam is proportional to

5 $$\begin{array}{rcl}{I}_{{\rm{LH}}/{\rm{RH}}} & = & \frac{{I}_{\hat{{\bf{p}}}}}{2{\omega }_{{\rm{L}}}^{2}}(({\rm{\Delta }}{\omega }^{2}+{({\rm{\Gamma }}/2)}^{2})\,{\cos }^{2}(\theta )\\  &  & \pm \,2{\rm{\Delta }}\omega {\omega }_{{\rm{L}}}\,\cos (\theta )+{\omega }_{{\rm{L}}}^{2})+\frac{{I}_{{\rm{perp}}}}{2}\,{\sin }^{2}(\theta )\end{array}$$

where *θ* is the angle of the magnetic field (which is defined to be in the $\hat{{\bf{z}}}$ direction) relative to $\hat{{\bf{k}}}$, and the upper/lower sign is used to find the intensity scattered by left-/right-handed polarized light. If the same intensity of pump light is used, the proportionality constant is the same for Eqs 1, 2 and 5. Otherwise, these equations must be scaled accordingly. Normalizing ${I}_{\hat{{\bf{p}}}}$ to ${I}_{{\rm{circ}}}=({I}_{{\rm{LH}}}+{I}_{{\rm{RH}}})$/2 results in the curves shown in Fig. 3(b). Additional details related to the derivation of these equations is given in the Supplementary Information.

## Results

**Figure 4:**
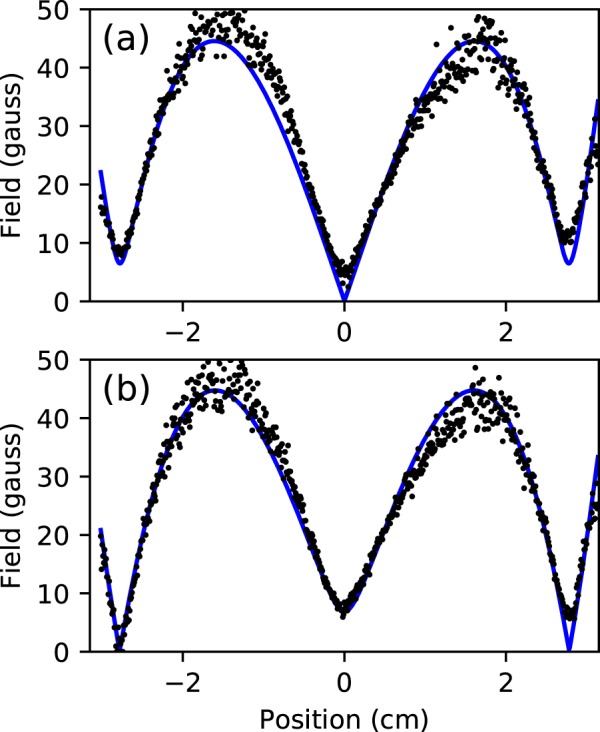
Magnetic field measurement. Using the full model normalized to data taken using circularly polarized light, the magnetic field along two horizontal lines of an image similar to the lower image in Fig. 1 was determined. The measured field is shown as dots. The solid line is the expected field for a pair of opposing ring magnets. Plot (**a**) shows the field along a horizontal line passing through the center of the central field zero. In (**b**) a similar plot is shown for a horizontal row of data displaced 0.85 mm vertically from the data used for the plot shown in (**a**).

The magnetic field along two horizontal lines, measured using the full quantum model, are shown in Fig. 4, along with the field we predicted using the known geometry of the magnets generating the field. The only free parameters used to fit the model to our measurements were the size of the effective surface currents (the *BHc* coercive field force) of the two magnets, and a 4% adjustment to the effective size we had estimated for the pixels in the images. Note that because the slope of the solid line in Fig. 3(a) is quite flat for fields much larger than *B*_HWHM_, for these fields small changes in measured light intensity correspond to large changes in magnetic field, such that small amounts of noise in the intensity measurements result in large errors in the measured field. This accounts for the increased scatter of the data points in regions of Fig. 4 with larger fields.

The outer two minima in Fig. 4(a) do not go to zero because of a known slight mismatch in the *BHc* of the two ring magnets generating the field. This offsets the ring-shaped zero crossing axially from the central zero crossing. Figure 4(b) shows the predicted and measured field magnitude along a horizontal line displaced 0.85 mm vertically from the center of the central field minimum. At this vertical displacement, both the predicted and measured field drop close to zero at the locations of the outer field minima.

While the data fits the expected field shape extremely well, in order to make the predicted field match our measurements, we had to set *BHc* for the two magnets to 1191 and 1217 kA/m, slightly above the expected range of 860–955 kA/m for N42 neodymium magnets^31^. The 2% difference in the magnetization of the two magnets agrees with Hall probe measurements of the magnets. The horizontal field gradient at the central zero, as determined using the directional Hanle effect, is 41 gauss/cm. This is somewhat larger than the 30 ± 4 gauss/cm that we estimated based on individual measurements of each of the magnets with a Hall probe in a separate test assembly. It is possible that our measurements using the directional Hanle effect are off by this much, perhaps due to nonlinearity in the response function of our camera (an inexpensive web cam). But it is also reasonable to believe that the field we measured is larger than we had predicted. This could be due to a combination of possible errors in our estimation of the expected field. Misalignment of the probe, such that it wasn’t exactly on axis, or non-uniform magnetization, such that the field axis was tilted relative to the geometric axis of the magnets, could have affected our Hall probe measurements. Errors in measurement of the magnet separation or imperfect alignment of the magnets could have also been a factor. It is possible that the cutting, bending, and welding of the steel during the fabrication of the vacuum chamber changed its magnetic properties, resulting in larger than expected magnetization effects. We were not able to break vacuum to do an *in*-*situ* Hall probe measurement for comparison.

### Field direction

With a slight extension to this method, in addition to the magnitude, the local *direction* of the magnetic field can also be determined. This is done by first measuring the polarization of the scattered light emitted in the $\hat{{\bf{p}}}$ direction, and then noting the asymmetry in the light scattered at low fields when viewed from a direction displaced from the $\hat{{\bf{p}}}$ direction.

**Figure 5:**
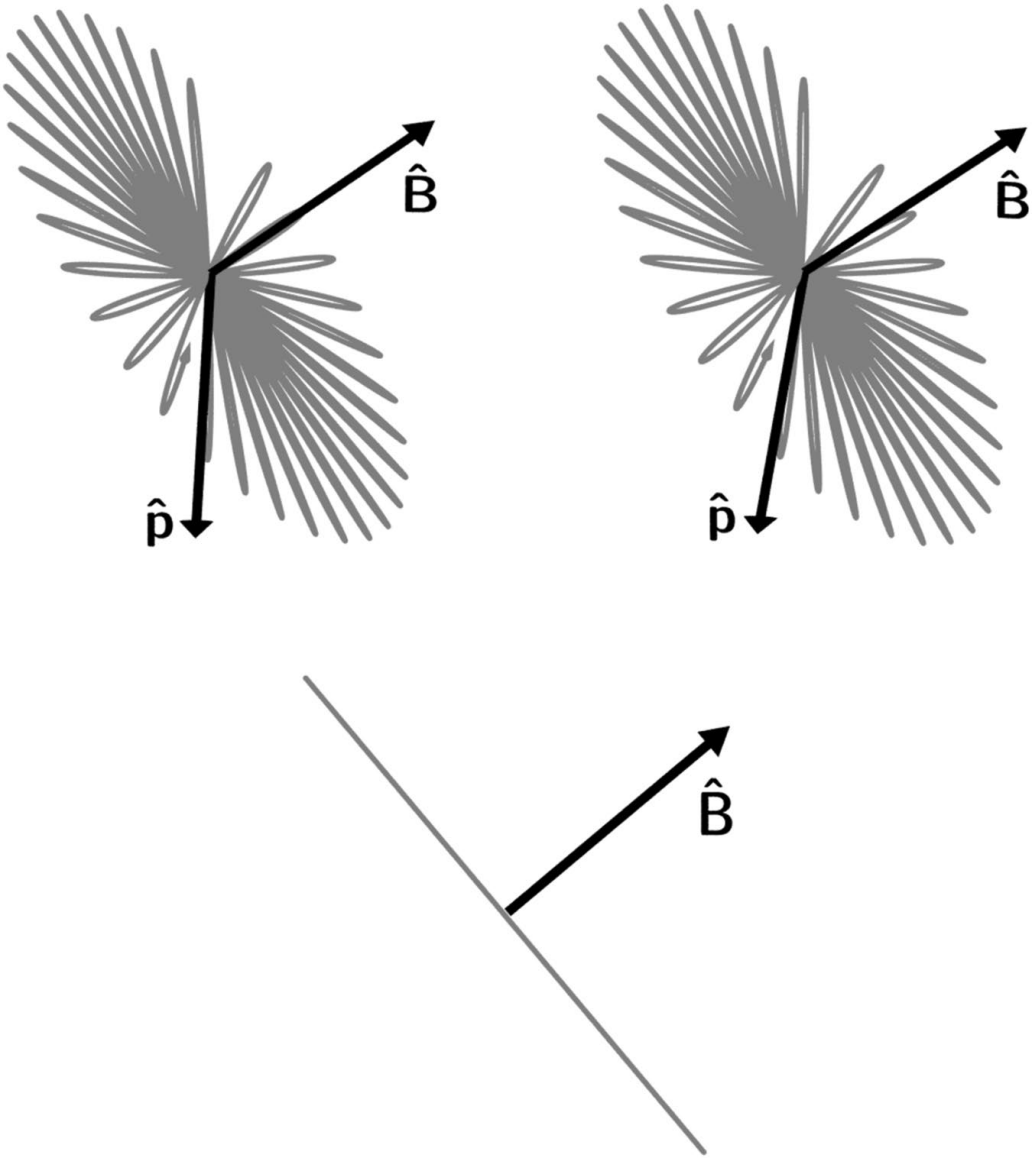
The polarization of the scattered light. The upper pair of images is a stereo pair for divergent ‘wall-eyed’ viewing. In each image the direction of the driving light polarization is indicated with the arrow labeled $\hat{{\bf{p}}}$. The magnetic field direction is shown with the arrow labeled $\hat{{\bf{B}}}$. A “cartoon” illustration of the path that the oscillating dipole moment takes is shown in gray. In the upper image pair, we see that the oscillation direction rotates in a plane orthogonal to the field direction. In the bottom image, we see the same path as viewed from a point along the line defined by $\hat{{\bf{p}}}$. From this vantage point, the projection of the oscillating dipole moment we see, and therefore the polarization of the light we measure, is always orthogonal to the field direction.

**Figure 6:**
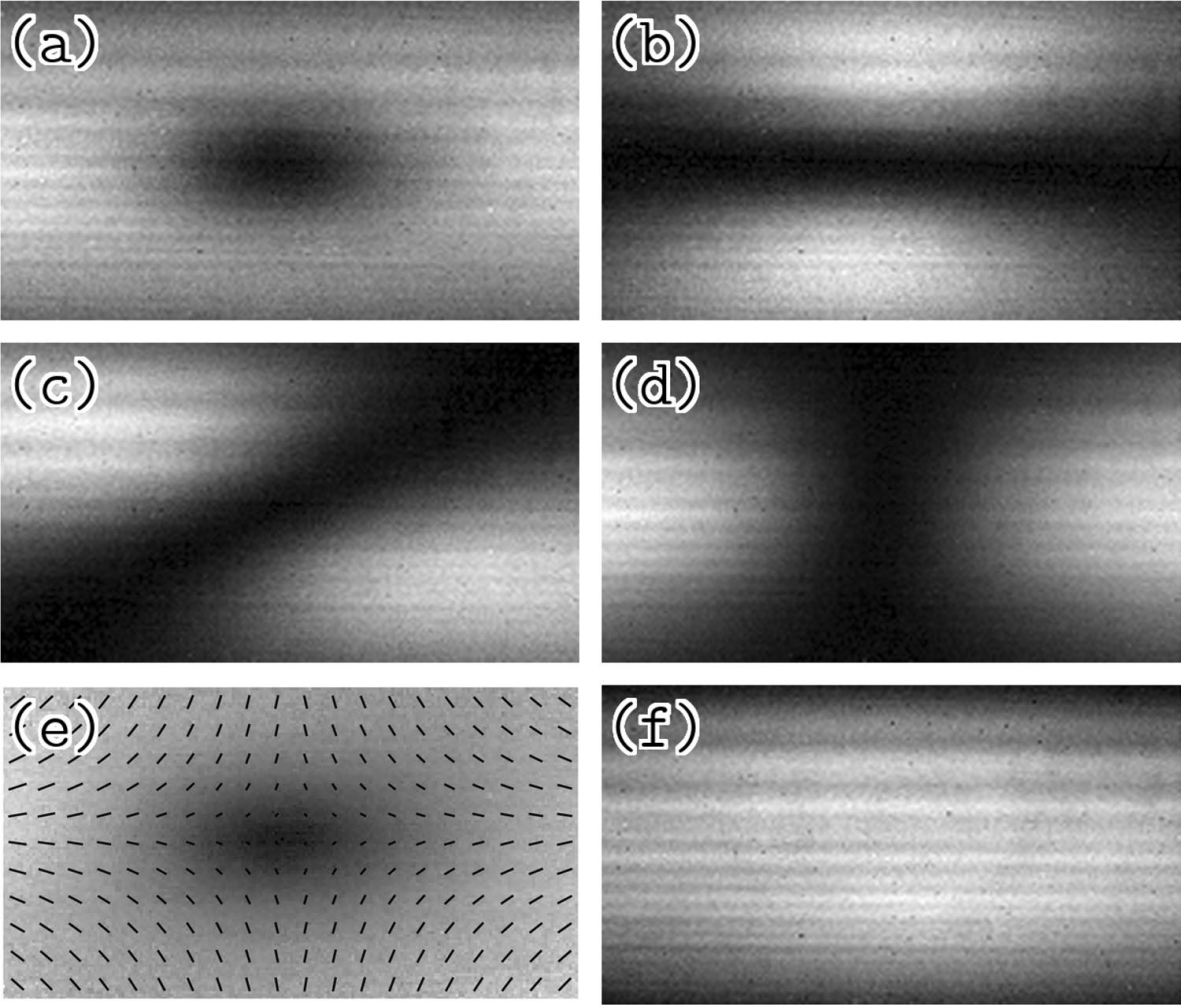
Images taken through a polarizer. Images of the fluorescing atoms were taken without a polarizer (**a**), and through a polarizer which allowed light to pass at a polarization angle of 0° (**b**), 45° (**c**), and 90° (**d**) from horizontal. The vector graph in (**e**) shows the direction of the field (to within an overall sign) calculated from the above images. Image (**f**) shows the average of two pictures taken with left- and right-handed circularly polarized light, in both cases without a polarizer in front of the camera. The horizontal stripes in the images are due to variations in pump light intensity across the light sheet.

If, as previously assumed, the magnetic field direction is perpendicular to $\hat{{\bf{p}}}$, the light scattered in the $\hat{{\bf{p}}}$ direction will be linearly polarized orthogonal to the local magnetic field. We can understand this intuitively by thinking of the oscillating atom as a classical oscillating dipole. The oscillation axis, initially excited along the $\hat{{\bf{p}}}$ direction, will rotate around the magnetic field, following a path similar to what is illustrated in Fig. 5. Knowing that the polarization of the light is perpendicular to the local field, the direction of the field (to within a sign) can be found using images such as those shown in Fig. 6(b–d), taken through a polarizer aligned to pass light polarized in different directions.

If $\hat{{\bf{B}}}$ is a unit vector in the direction of the field at a point in the images, the magnitudes of the components of $\hat{{\bf{B}}}$ are

6 $$|{\hat{{\bf{B}}}}_{h}|=\sqrt{\frac{{I}_{v}}{{I}_{h}+{I}_{v}}}\,{\rm{and}}\,|{\hat{{\bf{B}}}}_{v}|=\sqrt{\frac{{I}_{h}}{{I}_{h}+{I}_{v}}}$$

where *I*_*h*_ and *I*_*v*_ are the intensities at this point in the images taken using a horizontal and vertical polarizer, respectively. This narrows the direction of the field down to four possibilities: $\hat{{\bf{B}}}=\pm \,|{\hat{{\bf{B}}}}_{h}|\hat{{\bf{h}}}\pm |{\hat{{\bf{B}}}}_{v}|\hat{{\bf{v}}}$, where $\hat{{\bf{h}}}$ and $\hat{{\bf{v}}}$ are unit vectors in the horizontal and vertical directions. Using the image in Fig. 6(c), we can reduce this to two possibilities. The light intensity at this point in this image, *I*_*d*_, is related to the component of the magnetic field in the $(\hat{{\bf{h}}}+\hat{{\bf{v}}})$/$\sqrt{2}$ direction:

7 $$\frac{1}{\sqrt{2}}|{\hat{{\bf{B}}}}_{h}+{\hat{{\bf{B}}}}_{v}|=\sqrt{\frac{{I}_{d}}{{I}_{h}+{I}_{v}}}$$

From this, one can show that

8 $${\hat{{\bf{B}}}}_{h}{\hat{{\bf{B}}}}_{v}=\frac{{I}_{d}}{{I}_{h}+{I}_{v}}-\frac{1}{2}.$$

Calculating this we can determine the sign of ${\hat{{\bf{B}}}}_{h}{\hat{{\bf{B}}}}_{v}$ at each point in the image, which then tells us if the signs of the vertical and horizontal components of $\hat{{\bf{B}}}$ are the same or different. This gives us the direction of the magnetic field at this point, to within an overall sign. The results are shown in Fig. 6(e). Note that if the orthogonal 45° polarization axis is used, one side of the above equation must be multiplied by −1.

**Figure 7:**
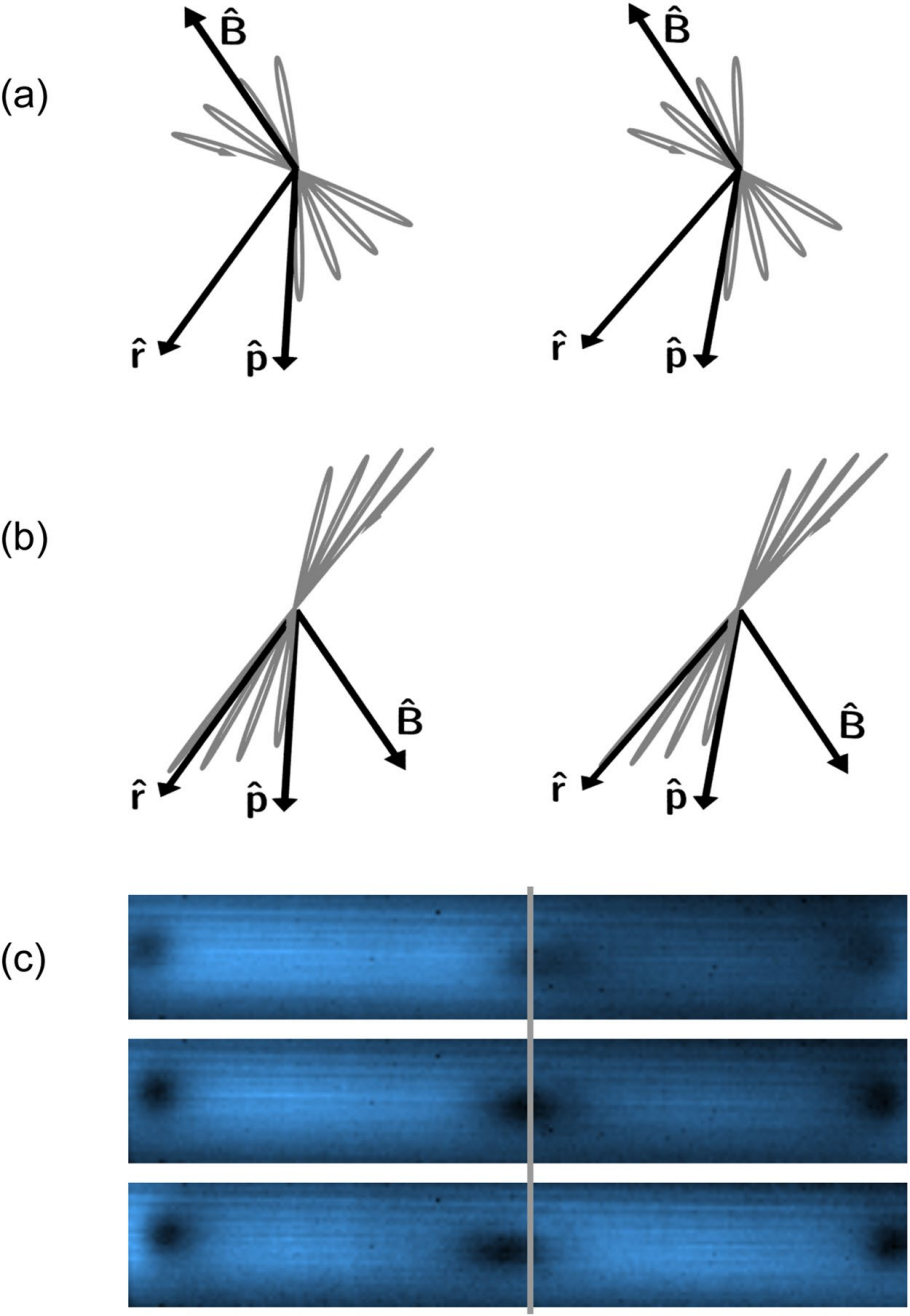
Scattered light asymmetry. Image pairs (**a**,**b**) are stereo pairs for divergent ‘wall-eyed’ viewing. In these images the black arrows show the direction from the atom to the camera (the $\hat{{\bf{r}}}$ direction), the direction of the driving light polarization direction ($\hat{{\bf{p}}}$) which is *not* parallel to $\hat{{\bf{r}}}$, and the direction of the magnetic field ($\hat{{\bf{B}}}$). The gray curve is a cartoon representation of the trajectory of the dipole moment. When the oscillating dipole is initially excited in the $\hat{{\bf{p}}}$ direction, there is a non-zero projection of the oscillation as seen by the camera. As shown in (**a**), the magnetic field could potentially rotate the dipole oscillation direction further away from $\hat{{\bf{r}}}$, increasing the light scattered to the camera. As shown in (**b**), if the field is in the other direction, the dipole oscillation direction rotates closer to $\hat{{\bf{r}}}$, decreasing the light scattered to the camera. The images in (**c**) were taken with the $\hat{{\bf{p}}}$ polarization direction rotated at different angles relative to the $\hat{{\bf{r}}}$ direction. The middle image was taken using light polarized in the $\hat{{\bf{r}}}$ direction. The upper/lower image was taken with the light polarization rotated 20° upward/downward from $\hat{{\bf{r}}}$. The gray line marks the horizontal center of the central field zero in the middle image. With the polarization rotated upward from $\hat{{\bf{r}}}$, light levels decrease relative to the same point in the $\hat{{\bf{p}}}=\hat{{\bf{r}}}$ picture if the field is pointed to the right, and increase if it is pointed to the left. With the polarization rotated downward, light levels decrease relative to the $\hat{{\bf{p}}}=\hat{{\bf{r}}}$ image if the field points to the left, and increase if it points to the right.

To determine the sign of the field at certain locations, we can take an image without a polarizer in front of the camera, but with the camera location offset from the $\hat{{\bf{p}}}$ direction. This can be done by moving the camera (such that $\hat{{\bf{r}}}$ is no longer parallel to $\hat{{\bf{x}}}$) or rotating the polarization of the driving light (such that $\hat{{\bf{p}}}$ is no longer parallel to $\hat{{\bf{x}}}$ — see Fig. 2). When this is done, the projection of the driving light polarization onto the plane of the camera will have a non-zero length, such that even in the absence of a magnetic field, some light will be scattered to the camera. If we think in terms of the classical oscillating dipole model, in regions of low field, such that the oscillation axis of the dipole only rotates a small amount before the oscillation has decayed, the magnetic field can cause the dipole oscillation axis to rotate even further from the camera’s line of sight, as shown in Fig. 7(a), increasing the light scattered to the camera. But, if the field is in the opposite direction, it can cause the dipole oscillation axis to rotate *toward* the camera, as shown in Fig. 7(b), making the light scattered to the camera go *down*.

**Figure 8:**
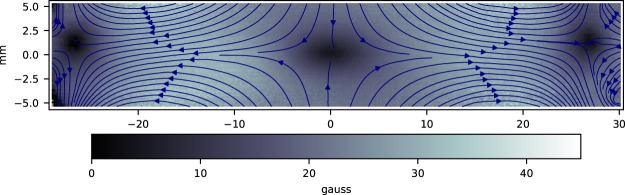
Magnetic field magnitude and direction. The direction of the magnetic field at different points in the image, determined using the images in Figs 6 and 7(c), is shown, along with the magnitude of the field at each point.

Images of the fluorescence, taken with the $\hat{{\bf{p}}}$ axis rotated relative to $\hat{{\bf{r}}}$, are shown in Fig. 7(c). One can infer the sign of the vector field shown in Fig. 6(e) from the asymmetry in these images. Because the dark region in the top image in Fig. 7(c) is displaced to the right, when compared to the middle image, we know that the field to the right of the dark region is in the +*k* direction, such that it rotates the oscillating dipole axis *toward* the camera, *reducing* the amount of light detected. The field at the left of the dark region is in the −*k* direction, such that it rotates the oscillating dipole axis *away* from the camera, *increasing* the amount of scattered light detected. Assuming that the field direction changes smoothly on the scale of a pixel, the direction of the field at other points can be inferred. Combining the information from this image with our other measurements, we can unambiguously determine both the magnitude and the direction of the field at each point in the image. The results are shown in Fig. 8.

## Discussion

In conclusion, we discussed and demonstrated a magnetic field measurement and visualization technique. The method, based on the directionality of scattered light in the Hanle effect, has some significant advantages for MOT alignment and field characterization. One of the most useful advantages is that it gives a direct visualization of the fields, including the location of field zero crossings and an estimate of the size of the MOT trapping volume. It is most sensitive to field strengths typical of those found in MOTs, requires little or no additional equipment or setup, and does not require a physical probe in the vacuum (other than the atoms themselves). The method can measure both the strength and the direction of the field.

Best results are obtained using a full quantum model^11^, but we also presented a simplified semi-classical model which is fairly accurate under certain conditions, and which is useful to build intuition for the directional Hanle effect. We also discussed methods to calibrate these measurements without the need to know the atomic density or pump light intensity at any point in the measurement region. Finally, we demonstrated this technique by measuring the magnitude and direction of the magnetic field at each point in a region of a plane passing through the center of a strontium MOT’s trapping volume.

## Methods

Most of the details of our experimental methods have already been discussed in the main text. We point out here some additional details about our apparatus and methods used to collect and analyze data.

The magnetic fields in our MOT are generated by a pair of N42 neodymium ring magnets. The rings have an inner/outer diameter of 5.08 cm (2 inches) and 7.62 cm (3 inches), respectively, and a 1.27 cm (1/2 inch) axial thickness. They are displaced from each other by 7.5 cm in the axial direction, and they are oriented such that their fields cancel at a point midway between them. One can think of a ring magnet as a thin sheet of current running along the inner surface of the ring, and an opposing sheet of current running along the ring’s outer diameter, such that each ring magnet is equivalent to two electromagnet coils with currents of equal magnitude running in opposite directions.

The two magnets together make a field approximating a spherical quadrupole field near the midpoint between the magnets along the symmetry axis. A ring-shaped zero crossing can occur, depending on the dimensions and separation of the rings, displaced radially from the central field zero. Early field measurements showed an asymmetry, probably due to a magnet that had been weakened when accidentally overheated (see the upper image in Fig. 1). The data analyzed in this paper was taken after this magnet was replaced to create a significantly less asymmetric field (see the lower image in the same figure).

It is not important which beam is used to illuminate the atoms. When using this technique to align MOT laser beams to the central field zero crossing, only the beam currently being aligned should be incident on the atoms. For the data presented and analyzed in this paper, the unblocked beam was a 461 nm laser beam used to slow atoms entering the trap from an oven. Because the light in this beam was initially circularly polarized, a linear polarizer was placed in the beam, and the polarization adjusted such that it was in a direction $\hat{{\bf{p}}}$ which was normal to the surface of the light sheet.

The camera we used to take our data was an inexpensive Logitech QuickCam Pro 9000 webcam with internal processing disabled^32,33^. With internal processing disabled, the pixel values are proportional to the intensity at each pixel, and we have access to the individual pixels of each color. Because the 461 nm laser light used in the experiment mainly registers on the blue pixels, we selected only those pixels for our analysis.

The driving light in our experiments had a peak saturation parameter of about 0.09 in the center of the pump beam. With the camera we used, it was difficult to collect good data with a weaker beam. For our analysis, we assumed the low saturation limit. This avoided the problem of needing to know how the intensity of driving light changed across the laser beam profile. Using a quantum model that includes optical saturation effects, we determined that along the center of the image, where the intensity is the brightest and errors from this assumption are the largest, this would produce errors in the measured magnetic field on the order of 10 percent. Incidentally, optical saturation effects are too small and in the wrong direction to account for the larger than expected magnetization fit parameters and field gradients discussed in the results section.

It is also worth noting that while the simplified model presented in the manuscript used a semi-classical approach, a fully classical model can be derived as well. While less intuitive and somewhat more difficult to derive than the semi-classical model, the fully classical model is not difficult to derive and yields the same results. The fully classical model can naturally be extended to a continuous rather than an impulse excitation. Furthermore, the continuous excitation classical model results in precisely the same equation as the full quantum model in the low intensity limit, given in Eq. 1.

We chose to supplement the quantum master equation model with a semi-classical, impulse excitation description, similar to what is traditionally used to introduce the Hanle effect^1^, rather than the fully classical model, because we feel that it gives a more intuitive picture, involving two beating terms generated by excited states, whose energy (and therefore frequency) differ due to Zeeman shifts. And, unlike the fully classical model, it can be easily extended to atoms with a more complicated excited state structure. Moreover, as the results of the classical continuous drive model are precisely Eq. 1, they don’t give us additional insight. The Lorentzian that results from the semi-classical model, however, is a very familiar and easy to interpret function, making it apparent what the approximate shape of the full curve should be for the conditions under which the semi-classical model is a reasonable approximation, and clearly revealing the field at which the Hanle effect saturates.

A full derivation of all of the models discussed in this paper, including the quantum master equation model, the simplified semi-classical model, and the fully classical model (with both impulse and continuous drive), is presented in the Supplementary Information.

## Supplementary information

Quantum, semi-classical, and classical models of the directional Hanle effect

## Data Availability

The datasets generated during and/or analyzed during the described study are available from the corresponding author on reasonable request.
