# Supplementary material for: Magneto-Optical Trap Field Characterization with the Directional Hanle Effect
Source: Sci Rep. 2019 Jun 20;9:8896. doi: 10.1038/s41598-019-45324-7 (PMC6586866; doi:10.1038/s41598-019-45324-7)
Supplement: Supplementary file 1 — Quantum, semi-classical, and classical models of the directional Hanle effect [file 41598_2019_45324_MOESM1_ESM.pdf]

Quantum, semi-classical, and classical models of the directional Hanle effect

A supplement to
"Magneto-optical trap field characterization with the directional Hanle effect"
by Jarom S. Jackson and Dallin S. Durfee

May 16, 2019

In this supplementary document, we give further information about the derivation of the quantum master equation and the semi-classical models of the directional Hale effect described in the paper "Magneto-optical trap field characterization with the directional Hanle effect," as well as the derivation of a fully classical model of the effect. Throughout this document we will refer to the paper, "Magneto-optical trap field characterization with the directional Hanle effect," as simply, "the manuscript."

These derivations apply specifically to a $j = 0$ ground state to a $j = 1$ excited state transition. The master equation approach mirrors what has been done in [1] for the conventional, polarization aspect of the Hanle effect. As such, similar calculations, mirroring calculations for the conventional Hanle effect, should be possible (though potentially much more complicated) for other types of transitions, including transitions involving multiple ground states, dark states, optical pumping, etc. In all of the models we assume that the probe field is not large enough for inelastic scattering. We will assume that the vapor is optically thin, as is the case for our apparatus, such that we don't have to consider absorption or re-scattering of scattered light. We assume atoms are driven by a thin sheet of light, that within the light sheet the local magnetic field direction, $\hat{z}$, lies within the plane of the sheet, and that the camera is displaced from the atoms in the direction normal to the surface of the light sheet. These assumptions are reasonable approximations for the experimental data presented in the manuscript.

1 Quantum master equation model

The model we used for all of the data analysis in the manuscript was derived using a master equation, similar to what is done in [1]. To consider other polarizations of light (so we could find I_{LH} and I_{RH} in Eq. 5 of the manuscript), we had to expand the treatment in [1] to include coupling to the $j = 1$, $m = 0$ sublevel. This derivation involves 4×4 matrices, and would be very tedious to do by hand. As such, we used the computer algebra tool *Mathematica* to assist in these calculations. The complete *Mathematica* code we used is listed in appendix A of this document. In this section we will briefly map out what is done in this code.

In the code we use two Cartesian coordinate systems. In Fig. 2 of the manuscript, there is a field-based Cartesian coordinate system defined such that $\hat{z}$ is in the direction of the local magnetic field, and $\hat{x}$ is defined to be perpendicular to the light sheet. A third direction, $\hat{y}$, is defined by $\hat{y} = \hat{z} \times \hat{x}$. In a second, lab-based coordinate system, $\hat{k}$ is the direction of the driving light propagation, and $\hat{x}$ is the direction perpendicular to the light sheet, the same direction as $\hat{x}$ in the field-based coordinate system. We introduce a third Cartesian coordinate in the lab frame, $\hat{d}$, or "down," not shown in Fig. 2 of the manuscript, which is defined by $\hat{d} = \hat{k} \times \hat{x}$. The angle between the laser beam propagation direction $\hat{k}$ and the field direction $\hat{z}$ is defined to be θ, such that $\hat{k} \times \hat{z} = \hat{d} \times \hat{y} = \hat{x} \sin(\theta)$.

We start with a Hamiltonian $H = H_0 + H_P$, which is the sum of the Hamiltonian for an atom in a magnetic field of magnitude B in the absence of the driving light, and a second term describing the interaction of the

atom with the driving light. We will let an atomic state be represented by a vector

$$|\psi\rangle = \begin{pmatrix} A_+ \\ A_- \\ A_0 \\ A_g \end{pmatrix}, \tag{1}$$

where A_+, A_-, and A_0 represent the amplitudes of the $m = 1$, $m = -1$, and $m = 0$ excited states, respectively, and A_g represents the amplitude of the $m = 0$ ground state. With this formalism,

$$H_0 = \hbar\omega_0 \begin{pmatrix} 1 & 0 & 0 & 0 \\ 0 & 1 & 0 & 0 \\ 0 & 0 & 1 & 0 \\ 0 & 0 & 0 & 0 \end{pmatrix} + \hbar\omega_{\mathrm{L}} \begin{pmatrix} -1 & 0 & 0 & 0 \\ 0 & 1 & 0 & 0 \\ 0 & 0 & 0 & 0 \\ 0 & 0 & 0 & 0 \end{pmatrix}, \tag{2}$$

where ω_0 is the resonance frequency for the $j = 0$ to $j = 1$ transition, and ω_{L} is the Larmor frequency

$$\omega_L = \frac{g\mu_{\mathrm{B}}B}{\hbar}, \tag{3}$$

with μ_{B} being the Bohr magneton, and g the Landé g-factor ($g = 1$ for the 5s5p $^1\mathrm{P}_1$ state in strontium).

The interaction with the driving light adds another term. To find this term, the code in appendix A starts with the polarization of light in the xdk lab frame. It then transforms the polarization into the xyz field frame. The z component couples the ground state to the $m = 0$ state. The code rewrites the x and y polarization components in terms of two circular polarizations about the z axis, each of which couples to one of the $m = \pm 1$ states. For our standard measurements, we have light oscillating at an angular frequency ω polarized in the $\hat{x}$ direction, such that our polarization vector (P_x, P_d, P_k) is $(1, 0, 0)$. In the field frame, the polarization vector (P_x, P_y, P_z) is equal to $(1, 0, 0)$ (as the x direction is the same for both frames). Writing the x and y polarizations in terms of circular polarizations, we find that the polarization vector (P_+, P_-, P_z) is equal to $(1/\sqrt{2}, -1/\sqrt{2}, 0)$. As such, the term in the Hamiltonian which describes the light field coupling the different states is

$$H_p = \frac{\hbar v}{\sqrt{2}} \begin{pmatrix} 0 & 0 & 0 & e^{i\omega t} \\ 0 & 0 & 0 & -e^{i\omega t} \\ 0 & 0 & 0 & 0 \\ e^{-i\omega t} & -e^{-i\omega t} & 0 & 0 \end{pmatrix}, \tag{4}$$

where ν is a constant proportional to the amplitude of the oscillating electric field.

For light which is circularly polarized, propagating in a direction $\hat{k}$, we start with a polarization in the lab frame of (P_x, P_d, P_k)= $(i/\sqrt{2}, \pm 1/\sqrt{2}, 0)$, where the upper/lower sign is for right-/left-handed circular polarization. Transforming into the field frame, assuming that the field direction z is at an angle θ relative to the k direction, we find that (P_x, P_y, P_z)= $(i/\sqrt{2}, \pm\cos(\theta)/\sqrt{2}, \pm\sin(\theta)/\sqrt{2})$, such that (P_+, P_-, P_z)= $(i(1 \pm \cos(\theta))/2, -i(1 \mp \cos(\theta))/2, \pm\sin(\theta)/\sqrt{2})$. This gives a Hamiltonian interaction term of

$$H_p = \hbar v \begin{pmatrix} 0 & 0 & 0 & \frac{i}{2}(1 \pm \cos(\theta)\, e^{i\omega t} \\ 0 & 0 & 0 & -\frac{i}{2}(1 \mp \cos(\theta)\, e^{i\omega t} \\ 0 & 0 & 0 & \pm\frac{1}{\sqrt{2}}\sin(\theta) e^{i\omega t} \\ -\frac{i}{2}(1 \pm \cos(\theta)\, e^{-i\omega t} & \frac{i}{2}(1 \mp \cos(\theta)) e^{-i\omega t} & \pm\frac{1}{\sqrt{2}}\sin(\theta) e^{-i\omega t} & 0 \end{pmatrix}. \tag{5}$$

Adding H_0 to the appropriate interaction term H_p for the chosen driving light polarization, we get the full Hamiltonian. We can then use the Hamiltonian to find the time evolution of the density matrix σ:

$$\frac{\partial\sigma}{\partial t} = -\frac{i}{\hbar}[H, \sigma], \tag{6}$$

where $[H, \sigma]$ is the commutator of the Hamiltonian and the density matrix. We will define the right hand of Eq. 6 to be Q:

$$Q = -\frac{i}{\hbar}[H, \sigma]. \tag{7}$$

In steady state, the density matrix will only undergo trivial changes related to the linear time evolution of the phases of atomic states. By going to an interaction-type of representation, we can remove this time dependence entirely. This is done by defining a new matrix $\hat{\rho}$ which is equal to $\hat{\sigma}$, except with the right-most column coherences between the ground and excited states multiplied by $e^{-i\omega t}$ and bottom row coherences multiplied by $e^{i\omega t}$. This gives us the matrix

$$\hat{\rho} = \begin{pmatrix} \rho_{++} & \rho_{-+} & \rho_{0+} & \rho_{g+} \\ \rho_{+-} & \rho_{--} & \rho_{0-} & \rho_{g-} \\ \rho_{+0} & \rho_{-0} & \rho_{00} & \rho_{g0} \\ \rho_{+g} & \rho_{-g} & \rho_{0g} & \rho_{gg} \end{pmatrix} = \begin{pmatrix} \sigma_{++} & \sigma_{-+} & \sigma_{0+} & \sigma_{g+}e^{-i\omega t} \\ \sigma_{+-} & \sigma_{--} & \sigma_{0-} & \sigma_{g-}e^{-i\omega t} \\ \sigma_{+0} & \sigma_{-0} & \sigma_{00} & \sigma_{g0}e^{-i\omega t} \\ \sigma_{+g}e^{i\omega t} & \sigma_{-g}e^{i\omega t} & \sigma_{0g}e^{i\omega t} & \sigma_{gg} \end{pmatrix}. \tag{8}$$

This transformation of the density matrix changes the time derivatives of the ground state coherences. For example the time derivative of ρ_{g+} is given by

$$\frac{\partial \rho_{g+}}{\partial t} = \frac{\partial}{\partial t}(\sigma_{g+}e^{-i\omega t}) = -i\omega\sigma_{g+}e^{-i\omega t} + \frac{\partial \sigma_{g+}}{\partial t}e^{-i\omega t} = -i\omega\rho_{g+} + e^{-i\omega t}\frac{\partial \sigma_{g+}}{\partial t}, \tag{9}$$

and

$$\frac{\partial \rho_{+g}}{\partial t} = \frac{\partial}{\partial t}(\sigma_{+g}e^{i\omega t}) = i\omega\sigma_{+g}e^{i\omega t} + \frac{\partial \sigma_{+g}}{\partial t}e^{i\omega t} = i\omega\rho_{+g} + e^{i\omega t}\frac{\partial \sigma_{+g}}{\partial t}, \tag{10}$$

In practice, we calculated Q, then replaced σ_{gq} and σ_{qg} (with q taking on the values $+$, $-$, and 0), wherever they appeared in Q, with $\rho_{gq}e^{i\omega t}$ and $\rho_{qg}e^{-i\omega t}$. Then, to account for the altered derivative, we multiplied Q_{qg} by $1/e^{i\omega t} + i\omega$, and multiplied Q_{gq} by $1/e^{-i\omega t} - i\omega$. This modified matrix then represents the time derivative $\partial\rho/\partial t$, which should be zero in steady state.

After doing this transformation, spontaneous decay can be added manually to the $\partial\rho/\partial t$ matrix, similar to what is done in [1]:

$$\frac{\partial \rho}{\partial t} \to \frac{\partial \rho}{\partial t} + \Gamma \begin{pmatrix} -\rho_{++} & -\rho_{-+} & -\rho_{0+} & -\rho_{g+}/2 \\ -\rho_{+-} & -\rho_{--} & -\rho_{0-} & -\rho_{g-}/2 \\ -\rho_{+0} & -\rho_{-0} & -\rho_{00} & -\rho_{g0}/2 \\ -\rho_{+g}/2 & -\rho_{-g}/2 & -\rho_{0g}/2 & \rho_{++} + \rho_{mm} + \rho_{00} \end{pmatrix}, \tag{11}$$

where Γ is the natural linewidth of the transition. Next, because ρ is Hermitian, we replaced ρ_{+-} with ρ_{-+}^*, ρ_{+0} with ρ_{0+}^*, etc., to reduce the number of unknown variables from 16 to 10. We then produce a set of equations to solve by setting $\partial\rho/\partial t$ to zero. Because of the symmetry of the matrix, the equations created by the lower triangle of $\partial\rho/\partial t$ are redundant with those from the upper triangle, such that this results in only 10 equations, equal to the number of unknowns. However, because of the relationships between the coherences and the diagonal terms in ρ, the 10 equations are not completely linearly independent. As such, we dropped one of the equations along the diagonal of $\partial\rho/\partial t$ and added the normalization constraint that $\rho_{++} + \rho_{--} + \rho_{00} + \rho_{gg} = 1$. This gives enough unique information such that this set of equations can be solved for the different components of ρ (ρ_{++}, ρ_{-+}, etc.) in steady state. To aid the computer in finding the solutions, we specifically divided each of the coherences into a real and imaginary part, and forced both the real and imaginary parts of each equation to zero (essentially turning the problem into one with 16 equations and 16 unknowns).

Once we have solved for the elements of ρ, we can find something proportional to the intensity of light emitted in different directions. For example, light emitted in the $\hat{x}$ direction consists of light polarized in the $\hat{z}$ direction emitted by the $m = 0$ upper state, plus a superposition of light emitted by the $m = \pm 1$ states which is polarized in the $\hat{y}$ direction. As such, the light emitted in the $\hat{x}$ direction is proportional to

$$|A_+ + A_-|^2 + 2|A_0|^2 = A_+A_+^* + A_-A_-^* + A_+A_-^* + A_-A_+^* + 2A_0A_0^* \tag{12}$$

$$= \rho_{++} + \rho_{--} + \rho_{+-} + \rho_{-+} + 2\rho_{00} \tag{13}$$

$$= \rho_{++} + \rho_{--} + 2\mathrm{Re}(\rho_{-+}) + 2\rho_{00}, \tag{14}$$

similar to Eq. 1 in [1], but with the addition of emission from the $m = 0$ state (which was not considered in [1] because this state is not excited by the polarization of light it assumed). Similarly, the light emitted in

the $\hat{y}$ direction is proportional to

$$|A_+ - A_-|^2 + 2|A_0|^2 = A_+A_+^* + A_-A_-^* - A_+A_-^* - A_-A_+^* + 2A_0A_0^* \tag{15}$$
$$= \rho_{++} + \rho_{--} - \rho_{+-} - \rho_{-+} + 2\rho_{00} \tag{16}$$
$$= \rho_{++} + \rho_{--} - 2\mathrm{Re}(\rho_{-+}) + 2\rho_{00}, \tag{17}$$

similar to Eq. 2 in [1], again with the addition of emission from the $m = 0$ state.

1.1 Linear polarization

For drive light linearly polarized in the $\hat{x}$ direction, Eq. 14 gives a fairly complicated expression:

$$\frac{32\omega_\mathrm{L}^2\nu^2\left(W + 2\nu^2\right)}{16\Delta\omega^4 W + 8\Delta\omega^2\left(\Gamma^4 + 6\nu^2 W - 16\omega_\mathrm{L}^4 + 2\nu^4\right) + \left(W + 2\nu^2\right)\left(2\nu^2\left(5\Gamma^2 + 4\omega_\mathrm{L}^2\right) + W^2 + 16\nu^4\right)}, \tag{18}$$

where

$$\Delta\omega = \omega - \omega_0 \tag{19}$$

and

$$W = \Gamma^2 + 4\omega_\mathrm{L}^2 \tag{20}$$

In the low saturation limit (i.e., to lowest order in ν), this becomes

$$I_{\hat{\mathbf{p}}} \propto \frac{2\omega_\mathrm{L}^2\nu^2}{\Delta\omega^4 + 2\Delta\omega^2\left((\Gamma/2)^2 - \omega_\mathrm{L}^2\right) + \left((\Gamma/2)^2 + \omega_\mathrm{L}^2\right)^2} \tag{21}$$

Similarly, in the absence of a magnetic field, for drive light linearly polarized in $\hat{x}$, Eq. 17 gives

$$\frac{8\nu^2\left(4\Delta\omega^2 W + \left(\Gamma^2 + 2\nu^2\right)\left(W + 2\nu^2\right)\right)}{16\Delta\omega^4 W + 8\Delta\omega^2\left(\Gamma^4 + 6\nu^2 W - 16\omega_\mathrm{L}^4 + 2\nu^4\right) + \left(W + 2\nu^2\right)\left(2\nu^2\left(5\Gamma^2 + 4\omega_\mathrm{L}^2\right) + W^2 + 16\nu^4\right)} \tag{22}$$

In the low saturation limit, in the absence of a magnetic field, this becomes

$$I_\mathrm{perp} \propto \frac{2\nu^2}{\Delta\omega^2 + (\Gamma/2)^2} \tag{23}$$

Taking the ratio of Eqs. 21 and 23, we get

$$\frac{I_{\hat{\mathbf{p}}}}{I_\mathrm{perp}} = \frac{\left[\Delta\omega^2 + (\Gamma/2)^2\right]\omega_\mathrm{L}^2}{\Delta\omega^4 + 2\Delta\omega^2\left((\Gamma/2)^2 - \omega_\mathrm{L}^2\right) + \left((\Gamma/2)^2 + \omega_\mathrm{L}^2\right)^2}, \tag{24}$$

as given in Eqs. 1 and 2 in the manuscript.

1.2 Circular polarization

For circularly polarized light, the solutions are much more complicated. However, in the limit of low saturation, the intensity emitted in the x direction is proportional to

$$I_\mathrm{LH/RH} \propto \frac{\nu^2\left(\left((\Gamma/2)^2 + \Delta\omega^2\right)\cos^2(\theta) \pm 2\Delta\omega\omega_\mathrm{L}\cos(\theta) + \omega_\mathrm{L}^2\right)}{\Delta\omega^4 + 2\Delta\omega^2\left((\Gamma/2)^2 - \omega_\mathrm{L}^2\right) + \left((\Gamma/2)^2 + \omega_\mathrm{L}^2\right)^2} + \frac{\nu^2\sin^2(\theta)}{2\left((\Gamma/2)^2 + \Delta\omega^2\right)} \tag{25}$$

such that

$$I_\mathrm{LH/RH} = \frac{I_{\hat{\mathbf{p}}}}{2\omega_\mathrm{L}^2}\left(\left(\Delta\omega^2 + (\Gamma/2)^2\right)\cos^2(\theta) \pm 2\Delta\omega\omega_\mathrm{L}\cos(\theta) + \omega_\mathrm{L}^2\right) + \frac{I_\mathrm{perp}}{2}\sin^2(\theta), \tag{26}$$

as given in Eq. 5 in the manuscript

2 Semi-classical model

The manuscript presents a simplified semi-classical model in order to help build intuition for the directional Hanle effect. While this model is not correct in the limit of high saturation, assumes an impulse rather than a continuous driving field, and involves many assumptions, we find it very useful for developing intuition for what physically happens in the directional Hanle effect. It illustrates how the Hanle effect can be thought of in terms of quantum beating, as discussed in [2] and [3]. In the semi-classical model we treat radiation fields classically, but quantize atomic energy levels.

After absorbing a photon, an atom's initial state will be a superposition of upper-state magnetic sublevels. The emissions from the different levels interfere, such that the total scattered field in a given direction depends on the relative phases of the emitting magnetic sublevels. Zeeman shifts split the degeneracy of the sublevels, causing their relative phases to change with time, altering the radiation field in a way analogous to the field of a classical oscillating dipole with a drifting oscillation axis.

For the assumed level structure, if the atom is excited to a superposition of excited-state magnetic sublevels, $|\Psi\rangle = a_0 |0\rangle + a_- |-1\rangle + a_+ |1\rangle$, the scattered light field in our model is the sum of classical oscillating dipole fields weighted by the amplitude coefficients a_0, a_-, and a_+. The magnitudes of these amplitudes after absorbing linearly polarized light depend on the angle of the polarization relative to the basis used to describe the atomic states, and are given by

$$|a_0| = \sqrt{\frac{R_0}{R_t}} \cos\theta_l \tag{27}$$

and

$$|a_\pm| = \sqrt{\frac{R_\pm}{2R_t}} \sin\theta_l, \tag{28}$$

where θ_l is the angle between the laser polarization and the z axis, and the parameter

$$R_{0,\pm} = \frac{I_L}{I_s}\left(\frac{\Gamma/2}{1 + I_L/I_s + 4\delta_{0,\pm}^2/\Gamma^2}\right), \tag{29}$$

is an off-resonance transition rate [4, 5] which depends on the transition linewidth Γ and the detuning $\delta_{0,\pm}$ (a sum of the laser offset from the zero-field resonance, Zeeman shifts, and Doppler shifts). I_L is the intensity of the driving light, I_s is the saturation intensity of the transition, and

$$R_t \equiv \cos^2\theta_l R_0 + \frac{1}{2}\sin^2\theta_l \left(R_+ + R_-\right). \tag{30}$$

The far-field radiation field $\mathbf{E}(\mathbf{r})$ at a displacement $\mathbf{r}$ from a classical dipole oscillating at an angular frequency ω and decaying according to a linewidth Γ is given by

$$\mathbf{E}(\mathbf{r}) = \frac{\omega^2}{4\pi\epsilon_0 c^2 r}(\hat{r} \times \mathbf{d}) \times \hat{r}\, e^{-\Gamma t_r/2} e^{-i\omega t_r} \tag{31}$$

(see [6]). Here ϵ_0 is the permittivity of free space, the time $t_r = t - r/c$ is the retarded time, and c is the speed of light. The vector $\mathbf{d}$ takes one of the values

$$\begin{aligned} \mathbf{d_0} &= d_0 \hat{z} && (m = 0) \\ \mathbf{d}_\pm &= \frac{1}{\sqrt{2}} d_0 \left(\hat{x} \pm i\hat{y}\right) && (m = \pm 1) \end{aligned} \tag{32}$$

to describe a dipole oscillating with an amplitude d_0 along the z axis (analogous to radiating from the $m = 0$ sublevel), or a dipole of magnitude $d_0/\sqrt{2}$ rotating around the z axis (analogous to radiating from the $m = \pm 1$ sublevels). The parameter d_0 can be calculated from the matrix element for the transition [7]. We assume all upper state sublevels decay with the same linewidth.

Zeeman shifts are typically minute compared to the atomic resonance frequency, so they will be neglected in the field amplitudes. But Zeeman shifts in the complex exponential factor in Eq. 31 cause the relative

phases of the field contributions to change in time, changing the radiation pattern, and generating the directional Hanle effect. We will define the direction of the local magnetic field to be z, and use the Zeeman shifted frequencies ω_0 and $\omega_\pm = \omega_0 \pm \Delta\omega$. Here $\Delta\omega = \mu_B g B/\hbar$, where $\hbar$ is Planck's constant over 2π, μ_B is the Bohr magneton, B is the field magnitude, and g is the Landé g-factor.

The total scattered field for a single atom is, then

$$\begin{aligned}\mathbf{E}_{\text{atom}}(\mathbf{r}) =& E_r e^{-\Gamma t_r/2}\Bigg[a_0\,(\hat{r}\times\hat{z})\times\hat{r}\,e^{-i\omega_0 t_r}\\ &+ a_+\left(\hat{r}\times\frac{1}{\sqrt{2}}(\hat{x}+i\hat{y})\right)\times\hat{r}\,e^{-i\omega_+ t_r}\\ &+ a_-\left(\hat{r}\times\frac{1}{\sqrt{2}}(\hat{x}-i\hat{y})\right)\times\hat{r}\,e^{-i\omega_- t_r}\Bigg],\end{aligned} \tag{33}$$

where $E_r = \omega_0^2 d_0/4\pi\epsilon_0 c^2 r$. We can find the field radiated by an ensemble of atoms by integrating the single atom intensity, $I_{\text{atom}} = \frac{1}{2}c\epsilon_0|\mathbf{E}_{\text{atom}}|^2$, over the distribution of Doppler-dependent transition rates and the retarded time (excited atoms are in different stages of decay).

At this point we will specifically consider the case of driving light polarized normal to the light sheet, i.e. in the direction from the atoms to the camera. With this assumption, the $m = 0$ upper-state sublevel is not populated. The combination of Doppler and Zeeman shifts can cause unequal transition rates to the $m = 1$ and $m = -1$ excited states. But to arrive at an analytical solution, in this simplified model we will assume that rates to the two sublevels are equal, and that these scattering rates are the same for every atom in the ensemble. In the case of broadband or strongly saturating driving light, this is approximately true. Even when it is not a good approximation, it allows us to find a simple analytical expression to aid intuition.

With these assumptions, Eq. 33 evaluated at the location of the camera can be simplified to

$$\mathbf{E}_{\text{atom}}(\mathbf{r}) = E_r \sin(\Delta\omega t_r)\, e^{-\Gamma t_r/2} e^{-i\omega_0 t_r}\hat{y}, \tag{34}$$

and the intensity radiated in the x direction is

$$I_{\text{atom}} = I_r \sin^2(\Delta\omega t_r) e^{-\Gamma t_r}, \tag{35}$$

where $I_r = c\epsilon_0 E_r^2/2$.

To get an ensemble-averaged intensity, we integrate this over t_r from 0 to ∞. The result is an inverted Lorentzian curve,

$$\langle I_{\text{ensemble}}\rangle = I_H\left[1 - \frac{1}{1+\frac{4\mu_B^2 g^2}{\hbar^2\Gamma^2}B^2}\right], \tag{36}$$

where I_H is a constant which depends on the driving light intensity and the density of atoms. This is presented as Eq. 3 in the manuscript.

3 Fully classical model with impulse excitation

As long as the linewidth of an atomic transition Γ is small compared to the resonant frequency ω_0, such that the atom undergoes many oscillations before a significant fraction of the excitation energy has been emitted, the Hanle effect for a $j = 0$ to $j = 1$ transition can be modeled classically as an under-damped harmonic oscillator. In the given approximations, these classical models exactly agree with the quantum master equation model and the simplified, intuitive semi-quantum impulse model given in the manuscript. In these fully classical derivations, we'll treat the oscillating dipole as a fixed positive charge harmonically bound to an oscillating electron with charge $-e$, a mass m, and a location $\vec{r}(t)$. We will first consider the simple case of a dipole excited with an impulse. We will then consider the steady state solution for a driven electric dipole.

If we assume that there are no external forces acting on the electron, its oscillation is determined by the equation of motion

$$\frac{d^2\vec{r}}{dt^2} + \omega_0^2\vec{r} + \Gamma\frac{d\vec{r}}{dt} = 0, \tag{37}$$

Defining $\vec{r}(t) = \hat{i}x(t) + \hat{j}y(t) + \hat{k}z(t)$, This equation can be separated into three scalar equations, each of the form

$$\frac{d^2x}{dt^2} + \omega_0^2 x + \Gamma\frac{dx}{dt} = 0 \tag{38}$$

The solutions to these equation are sinusoidal oscillations with a decaying amplitude:

$$x(t) = A_x e^{-\Gamma t/2}\sin(\omega_d t + \phi_x), \tag{39}$$

$$y(t) = A_y e^{-\Gamma t/2}\sin(\omega_d t + \phi_y), \tag{40}$$

$$z(t) = A_z e^{-\Gamma t/2}\sin(\omega_d t + \phi_z). \tag{41}$$

where $A_{x,y,z}$ and $\phi_{x,y,z}$ are constants and

$$\omega_d = \sqrt{\omega_0^2 - (\Gamma/2)^2}. \tag{42}$$

If a constant magnetic field is applied to this system, the equation of motion becomes

$$\frac{d^2\vec{r}}{dt^2} + \omega_0^2\vec{r} + \Gamma\frac{d\vec{r}}{dt} + \frac{e}{m}\frac{d\vec{r}}{dt} \times \vec{B} = 0. \tag{43}$$

Larmor's theorem tells us that if the field is weak enough, such that the Larmor frequency is much smaller than the atomic resonance frequency ω_0, to a good approximation the effect of the field can be removed by transforming into a reference frame rotating about the field direction at the Larmor frequency. We'll define the direction of the field to be z, and define the rotating reference frame variables

$$x_r = x\cos(\omega_\mathrm{L} t) - y\sin(\omega_\mathrm{L} t) \tag{44}$$

and

$$y_r = y\cos(\omega_\mathrm{L} t) + x\sin(\omega_\mathrm{L} t), \tag{45}$$

such that

$$x = x_r\cos(\omega_\mathrm{L} t) + y_r\sin(\omega_\mathrm{L} t) \tag{46}$$

and

$$y = y_r\cos(\omega_\mathrm{L} t) - x_r\sin(\omega_\mathrm{L} t). \tag{47}$$

If the dipole is initially excited in the x direction, which at $t = 0$ is equivalent to the x_r direction, the dipole will simply oscillate in the x_r direction:

$$x_r(t) = Ae^{-\Gamma t/2}\sin(\omega_0 t + \phi), \tag{48}$$

where A and ϕ are constants. Transforming back into the lab frame,

$$x(t) = Ae^{-\Gamma t/2}\sin(\omega_0 t + \phi)\cos(\omega_\mathrm{L} t) \tag{49}$$

and

$$y(t) = -Ae^{-\Gamma t/2}\sin(\omega_0 t + \phi)\sin(\omega_\mathrm{L} t). \tag{50}$$

As such, the instantaneous intensity scattered in the x direction would be proportional to

$$|y(t)|^2 = A^2 e^{-\Gamma t}\sin^2(\omega_0 t + \phi)\sin^2(\omega_\mathrm{L} t). \tag{51}$$

To get something proportional to the intensity averaged over many scattering events, which we call $\langle I_\mathrm{ensemble}\rangle$ in the manuscript, we integrate this from $t = 0$ to ∞. We have already assumed that $\omega_0 \gg \omega_\mathrm{L}$. If we further assume that $\omega_0 \gg \Gamma$ (such that the oscillation decays over many periods), we can approximate the $\sin^2(\omega_0 t + \phi)$ term as 1/2 in the integral, such that

$$\langle I_\mathrm{ensemble}\rangle \propto \int_0^\infty \frac{1}{2}A^2 e^{-\Gamma t}\sin^2(\omega_\mathrm{L} t)dt = \frac{A^2\omega_\mathrm{L}^2}{\Gamma^3 + 4\Gamma\omega_\mathrm{L}^2}. \tag{52}$$

This is to be compared to the intensity that would be scattered in a direction perpendicular to $\hat{x}$ in the absence of a magnetic field (which we call I_{perp} in the manuscript) which is proportional to

$$I_{\text{perp}} = \int_0^\infty \frac{1}{2} A e^{-\Gamma t/2} dt = \frac{A^2}{2\Gamma} \tag{53}$$

From this, we can write

$$I_{\hat{\mathbf{p}}} = I_{\text{perp}} \frac{2\omega_L^2}{\Gamma^2 + 4\omega_{\text{L}}^2} = \frac{I_{\text{perp}}}{2} \left[1 - \frac{1}{1 + \frac{4\mu_B^2}{\hbar^2 \Gamma^2} B^2} \right], \tag{54}$$

which is equivalent to Eq. 3 in the manuscript.

4 Fully classical driven, steady state model

We can also make a fully classical model in which we consider an atom which is continuously driven by an oscillating driving field. The equations become less intuitive and harder to solve in this case. As such, parts of the derivations presented below were done with the assistance of a computer algebra package. The code used is given in Appendices B and C.

4.1 Linearly polarized driving light

We will first consider a driving field polarized in the x direction, oscillating with a frequency $\omega = \omega_0 + \Delta\omega$, $\Delta\omega$ being the detuning from the atomic resonance frequency. In the absence of a magnetic field, the equations of motion are

$$\frac{d^2 x}{dt^2} + \omega_0^2 x + \Gamma \frac{dx}{dt} = F_0 e^{i(\omega_0 + \Delta\omega)t}, \tag{55}$$

$$\frac{d^2 y}{dt^2} + \omega_0^2 y + \Gamma \frac{dy}{dt} = 0, \text{ and} \tag{56}$$

$$\frac{d^2 z}{dt^2} + \omega_0^2 z + \Gamma \frac{dz}{dt} = 0, \tag{57}$$

where F_0 is a (potentially complex) constant. With no magnetic field, the steady state solutions to these equations are

$$x(t) = \frac{-F_0}{\Delta\omega^2 + \Delta\omega(2\omega_0 - i\Gamma) - i\Gamma\omega_0} e^{i(\omega_0 + \Delta\omega)t}, \tag{58}$$

$$y(t) = 0, \tag{59}$$

and

$$z(t) = 0. \tag{60}$$

If we assume that ω_0 is much larger than any of the other frequencies (δ, ω_L, and Γ), we can simplify this solution by expanding the amplitude of the oscillation in x in terms of powers of $1/\omega_0$, and keeping only the lowest order term. Doing that, we get

$$x(t) = \frac{-F_0/\omega_0}{2\Delta\omega - i\Gamma} e^{i(\omega_0 + \Delta\omega)t} \tag{61}$$

The intensity of light scattered in a direction orthogonal to x will be proportional to the magnitude of the oscillation in x squared:

$$I_{\text{perp}} \propto \left| \frac{-F_0/\omega_0}{2\Delta\omega - i\Gamma} \right|^2 = \left| \frac{-F_0/2\omega_0}{\Delta\omega - i\Gamma/2} \right|^2 = \frac{|F_0|^2/4\omega_0^2}{\Delta\omega^2 + (\Gamma/2)^2} \tag{62}$$

If we add a magnetic field in the z direction, the z equation is unchanged, such that in steady state $z(t)$ is still equal to zero. If we transform into the rotating frame, according to Larmor's theorem the first two equations become

$$\frac{d^2 x_r}{dt^2} + \omega_0^2 x_r + \Gamma \frac{dx_r}{dt} = F_0 \cos(\omega_{\text{L}} t) e^{i(\omega_0 + \Delta\omega)t} = \frac{F_0}{2} e^{i(\omega_0 + \Delta\omega + \omega_{\text{L}})t} + \frac{F_0}{2} e^{i(\omega_0 + \Delta\omega - \omega_{\text{L}})t} \tag{63}$$

and

$$\frac{d^2y_r}{dt^2} + \omega_0^2 y_r + \Gamma\frac{dy_r}{dt} = F_0\sin(\omega_{\rm L}t)e^{i(\omega_0+\Delta\omega)t} = -\frac{iF_0}{2}e^{i(\omega_0+\Delta\omega+\omega_{\rm L})t} + \frac{iF_0}{2}e^{i(\omega_0+\Delta\omega-\omega_{\rm L})t}. \tag{64}$$

If we assume steady state solutions for x_r and y_r of the form

$$x_r(t) = A_{x+}e^{i(\omega_0+\Delta\omega+\omega_{\rm L})t} + A_{x-}e^{i(\omega_0+\Delta\omega-\omega_{\rm L})t} \tag{65}$$

$$y_r(t) = A_{y+}e^{i(\omega_0+\Delta\omega+\omega_{\rm L})t} + A_{y-}e^{i(\omega_0+\Delta\omega-\omega_{\rm L})t}, \tag{66}$$

where $A_{x\pm}$ and $A_{y\pm}$ are complex constants, and plug them into Eqs. 63 and 64, we find that

$$A_{x+} = \frac{F_0/2}{-(\omega_0+\Delta\omega+\omega_{\rm L})^2 + \omega_0^2 + i\Gamma(\omega_0+\Delta\omega+\omega_{\rm L})}, \tag{67}$$

$$A_{x-} = \frac{F_0/2}{-(\omega_0+\Delta\omega-\omega_{\rm L})^2 + \omega_0^2 + i\Gamma(\omega_0+\Delta\omega-\omega_{\rm L})}, \tag{68}$$

$$A_{y+} = \frac{-iF_0/2}{-(\omega_0+\Delta\omega+\omega_{\rm L})^2 + \omega_0^2 + i\Gamma(\omega_0+\Delta\omega+\omega_{\rm L})}, \tag{69}$$

and

$$A_{y-} = \frac{iF_0/2}{-(\omega_0+\Delta\omega-\omega_{\rm L})^2 + \omega_0^2 + i\Gamma(\omega_0+\Delta\omega-\omega_{\rm L})}. \tag{70}$$

Transforming this solutions back into the lab frame results in something fairly complicated. But calculating $|y|^2$ in the limit that ω_0 is much larger than $\Delta\omega$ and $\omega_{\rm L}$, we get

$$I_{\hat{\mathbf{p}}} \propto \frac{\omega_{\rm L}^2 F_0^2/4\omega_0^2}{\Delta\omega^4 + 2\Delta\omega^2\left((\Gamma/2)^2 - \omega_{\rm L}^2\right) + \left((\Gamma/2)^2 + \omega_{\rm L}^2\right)^2} \tag{71}$$

Taking the ratio of this to Eq. 62, we get

$$\frac{I_{\hat{\mathbf{p}}}}{I_{\rm perp}} = \frac{\left[\Delta\omega^2 + (\Gamma/2)^2\right]\omega_{\rm L}^2}{\Delta\omega^4 + 2\Delta\omega^2\left((\Gamma/2)^2 - \omega_{\rm L}^2\right) + \left((\Gamma/2)^2 + \omega_{\rm L}^2\right)^2} \tag{72}$$

which is precisely equal to Eq. 24 derived using a quantum master equation approach in the low drive intensity limit.

4.2 Circularly polarized driving light

If we instead assume circularly polarized light traveling at an angle θ relative to the z axis, in the absence of a magnetic field the equations of motion are

$$\frac{d^2x}{dt^2} + \omega_0^2 x_r + \Gamma\frac{dx}{dt} = F_0\frac{1}{\sqrt{2}}e^{i(\omega_0+\Delta\omega)t}, \tag{73}$$

$$\frac{d^2y}{dt^2} + \omega_0^2 y + \Gamma\frac{dy}{dt} = \mp F_0\cos(\theta)\frac{i}{\sqrt{2}}e^{i(\omega_0+\Delta\omega)t}, \tag{74}$$

and

$$\frac{d^2z}{dt^2} + \omega_0^2 z + \Gamma\frac{dz}{dt} = \mp F_0\sin(\theta)\frac{i}{\sqrt{2}}e^{i(\omega_0+\Delta\omega)t}, \tag{75}$$

where the upper/lower sign corresponds to right-/left-handed polarization. Adding a magnetic field and then transforming into the rotating frame leaves the equation for z unchanged. It's solution should have the form of the driving field, such that

$$z(t) = A_z e^{i(\omega_0+\Delta\omega)t}. \tag{76}$$

Plugging this into Eq. 75, we find that

$$A_z = \frac{\mp iF_0\sin(\theta)/\sqrt{2}}{-2\omega_0\Delta\omega - \Delta\omega^2 + i\Gamma(\omega_0+\Delta\omega)}. \tag{77}$$

As such, the intensity of the component of light scattered in the x direction which has a polarization in z is proportional to

$$|A_z|^2 = \frac{|F_0|^2\sin^2(\theta)/2}{(\Delta\omega^2+2\omega_0\Delta\omega)^2+\Gamma^2(\omega_0+\Delta\omega)^2}. \tag{78}$$

If we assume that ω_0 is very large compared to $\Delta\omega$, this becomes

$$\frac{|F_0|^2\sin^2(\theta)/2}{4\omega_0^2\left(\Delta\omega^2+(\Gamma/2)^2\right)} = \frac{I_{\text{perp}}\sin^2(\theta)}{2} \tag{79}$$

The x and y equations in the rotating frame become

$$\frac{d^2x_r}{dt^2}+\omega_0^2x_r+\Gamma\frac{dx_r}{dt} = F_0\left[\cos(\omega_\text{L}t)\pm i\sin(\omega_\text{L}t)\cos(\theta)\right]\frac{1}{\sqrt{2}}e^{i(\omega_0+\Delta\omega)t}, \tag{80}$$

$$= \frac{F_0}{2\sqrt{2}}\left[e^{i\omega_\text{L}t}+e^{-i\omega_\text{L}t}\pm\left(e^{i\omega_\text{L}t}-e^{-i\omega_\text{L}t}\right)\cos(\theta)\right]e^{i(\omega_0+\Delta\omega)t} \tag{81}$$

$$= \frac{F_0}{2\sqrt{2}}\left[(1\pm\cos(\theta))\,e^{i(\omega_0+\Delta\omega+\omega_\text{L})t}+(1\mp\cos(\theta))\,e^{i(\omega_0+\Delta\omega-\omega_\text{L})t}\right] \tag{82}$$

and

$$\frac{d^2y_r}{dt^2}+\omega_0^2y+\Gamma\frac{dy}{dt} = F_0\left[\mp i\cos(\omega_\text{L}t)\cos(\theta)+\sin(\omega_\text{L}t)\right]\frac{1}{\sqrt{2}}e^{i(\omega_0+\Delta\omega)t} \tag{83}$$

$$= \frac{iF_0}{2\sqrt{2}}\left[\mp\left(e^{i\omega_\text{L}t}+e^{-i\omega_\text{L}\,t}\right)\cos(\theta)-e^{i\omega_\text{L}t}+e^{-i\omega_\text{L}t}\right]e^{i(\omega_0+\Delta\omega)t} \tag{84}$$

$$= \frac{iF_0}{2\sqrt{2}}\left[-(1\pm\cos(\theta))\,e^{i(\omega_0+\Delta\omega+\omega_\text{L})t}+(1\mp\cos(\theta))\,e^{i(\omega_0+\Delta\omega-\omega_\text{L})t}\right]. \tag{85}$$

Again, assuming steady state solutions of the form

$$x_r(t) = A_{x+}e^{i(\omega_0+\Delta\omega+\omega_\text{L})t}+A_{x-}e^{i(\omega_0+\Delta\omega-\omega_\text{L})t} \tag{86}$$

$$y_r(t) = A_{y+}e^{i(\omega_0+\Delta\omega+\omega_\text{L})t}+A_{y-}e^{i(\omega_0+\Delta\omega-\omega_\text{L})t}, \tag{87}$$

we find that

$$A_{x+} = \frac{F_0\left(1\pm\cos(\theta)\right)/2\sqrt{2}}{-(\omega_0+\Delta\omega+\omega_\text{L})^2+\omega_0^2+i\Gamma(\omega_0+\Delta\omega+\omega_\text{L})}, \tag{88}$$

$$A_{x-} = \frac{F_0\left(1\mp\cos(\theta)\right)/2\sqrt{2}}{-(\omega_0+\Delta\omega-\omega_\text{L})^2+\omega_0^2+i\Gamma(\omega_0+\Delta\omega-\omega_\text{L})}, \tag{89}$$

$$A_{y+} = \frac{-iF_0\left(1\pm\cos(\theta)\right)/2\sqrt{2}}{-(\omega_0+\Delta\omega+\omega_\text{L})^2+\omega_0^2+i\Gamma(\omega_0+\Delta\omega+\omega_\text{L})}, \tag{90}$$

and

$$A_{y-} = \frac{iF_0\left(1\mp\cos(\theta)\right)/2\sqrt{2}}{-(\omega_0+\Delta\omega-\omega_\text{L})^2+\omega_0^2+i\Gamma(\omega_0+\Delta\omega-\omega_\text{L})}. \tag{91}$$

Transforming our solutions back to the lab frame, and calculating $|y|^2$ with the assumption that ω_0 is very large compared to ω_L and $\Delta\omega$, we find that the intensity of the component of light scattered in the x direction which has a polarization in y is

$$\frac{I_{\hat{\mathbf{p}}}}{2\omega_\text{L}^2}\left(\left((\Gamma/2)^2+\Delta\omega^2\right)\cos^2(\theta)\pm2\Delta\omega\omega_\text{L}\cos(\theta)+\omega_\text{L}^2\right) \tag{92}$$

such that the total intensity scattered in the x direction is equal to

$$I_{\text{LH/RH}} = \frac{I_{\hat{\mathbf{p}}}}{2\omega_\text{L}^2}\left(\left((\Gamma/2)^2+\Delta\omega^2\right)\cos^2(\theta)\pm2\Delta\omega\omega_\text{L}\cos(\theta)+\omega_\text{L}^2\right)+I_{\text{perp}}\frac{\sin^2(\theta)}{2}, \tag{93}$$

in agreement with Eq. 26.

References

[1] Avan, P. and Cohen-Tannoudji, C. Hanle effect for monochromatic excitation. Non perturbative calculation for a $j = 0$ to $j = 1$ transition. *Le J. de Physique - Lett.* **36**, L85–L88 (1975).

[2] Budker, D., Gawlik, W., Kimball, D. F., Rochester, S. M., Yashchuk,V. V. and Weis, A. Resonant nonlinear magneto-optical effects in atoms. *Rev. Mod. Phys.* **74**, 1153–1201, (2002).

[3] Novikov, L. N., Skrotskii, G. V. and Solomakh, G. I. The Hanle effect. *Usp. Fiz. Nauk* **113**, 597–625, (1974).

[4] Metcalf, H. and van der Straten, P., Cooling and trapping of neutral atoms, *Physics Reports*, **244**, 203–286, (1994).

[5] Budker, D., Kimball, D. F. and DeMille, D. P., *Atomic Physics,* Oxford University Press, (2008).

[6] Jackson, J. D., *Classical Electrodynamics,* John Wiley and Sons (1975).

[7] Jackson, J. S., *In Situ Magnetic Field Characterization with the Directional Hanle Effect*, Masters Thesis, Brigham Young University, (2016).

A Mathematica code for the quantum master equation calculation

```
Quit[]

$Assumptions = Element[{G, hbar, Oe, wo, w, v, t, spp, smm, soo, sgg, pgpa, pgpb,
    pgma, pgmb, pgoa, pgob, smpa, smpb, sopa, sopb, soma, somb, theta}, Reals]

(G | hbar | Oe | wo | w | v | t | spp | smm | soo | sgg | pgpa | pgpb | pgma |
    pgmb | pgoa | pgob | smpa | smpb | sopa | sopb | soma | somb | theta) ∈ Reals

(* Define variables for each density matrix location *)

s = {{spp, smp, sop, sgp}, {smp*, smm, som, sgm},
    {sop*, som*, soo, sgo}, {sgp*, sgm*, sgo*, sgg}};

Grid[s]

      spp             smp             sop       sgp
Conjugate[smp]        smm             som       sgm
Conjugate[sop] Conjugate[som]         soo       sgo
Conjugate[sgp] Conjugate[sgm] Conjugate[sgo] sgg

(* Define variables for each interaction picture density matrix location *)

p = {{spp, smp, sop, pgp}, {smp*, smm, som, pgm},
    {sop*, som*, soo, pgo}, {pgp*, pgm*, pgo*, sgg}};

(* Make a list of the variables we will solve for *)

varlist = DeleteCases[
  Flatten[UpperTriangularize[p] /. smp → {smpa, smpb} /. sop → {sopa, sopb} /.
        som → {soma, somb} /. pgp → {pgpa, pgpb} /.
      pgm → {pgma, pgmb} /. pgo → {pgoa, pgob}], 0]

{spp, smpa, smpb, sopa, sopb, pgpa, pgpb,
 smm, soma, somb, pgma, pgmb, soo, pgoa, pgob, sgg}
```

```
(* Calculating the light scattered by an atom on a J=
 0 to J=1 transition in a constant magnetic field.

    First, our coordinate system:
  The magnetic field is in the z direction.  The light propogates in the k
   direction.  The z direction is at an angle theta to the k direction,
and the x direction is the direction where we normally place the camera,
such that x is perpendicular to k and z.  The y direction is
  orthogonal to x and z to form a right-handed coordinate system xyz.
   We'll define another direction d such that xdk make a right-
 handed coordinate system.  Since z is at an angle theta to k,
y is at an angle theta to d.  xdk form the lab coordinate system,
and xyz form the field coordinate system.

   There are four states: the m=+1, the m=-1, the m=0,
and the ground state (which is also m=0).  I'll define the
 state vector to have the order {+,-,0,g}.  If I define wo to be
 the natural resonance frequency of the transition and Oe to be
 the Zeeman frequency shift, such that Oe = mu_B * g * B/hbar,
and if I assume that the field is in the +z direction,
then the unperturbed Hamiltonioan is *)

Ho = hbar * {{wo - Oe, 0, 0, 0}, {0, wo + Oe, 0, 0}, {0, 0, wo, 0}, {0, 0, 0, 0}};

Grid[Ho]
```

hbar (-Oe + wo)	0	0	0
0	hbar (Oe + wo)	0	0
0	0	hbar wo	0
0	0	0	0

```
(* The light can be polarized in the x, d, or z directions,
or a superposition of the three with arbitrary phase *)

(* For example, for the effect we generally look at,
we place the camera in the x direction and point the polarization at the camera,
so we can set the polarization vector to P=
 {1,0,0}.  This is the first case we will consider. *)

Pxdk = {1, 0, 0}

{1, 0, 0}

(* Now we translate this into the atom coordinates *)

Pxyz = {Pxdk[[1]], Pxdk[[2]] * Cos[theta] - Pxdk[[3]] * Sin[theta],
  Pxdk[[3]] * Cos[theta] + Pxdk[[2]] * Sin[theta]}

{1, 0, 0}

(* We now write this in terms of circular polarizations *)
```

```
Pc = Simplify[{(Pxyz[[1]] + I * Pxyz[[2]]) / Sqrt[2],
   -(Pxyz[[1]] - I * Pxyz[[2]]) / Sqrt[2], Pxyz[[3]]}]
```

$$\left\{\frac{1}{\sqrt{2}}, -\frac{1}{\sqrt{2}}, 0\right\}$$

```
(* If v is proportional to the light amplitude and the dipole moment,
and the angular frequency of the light is w,
the perterbation on the Hamiltonian caused by the light is *)

Hp = hbar * v * {{0, 0, 0, Pc[[1]] * Exp[I * w * t]},
    {0, 0, 0, Pc[[2]] * Exp[I * w * t]}, {0, 0, 0, Pc[[3]] * Exp[I * w * t]},
    {Pc[[1]]\[Conjugate] * Exp[-I * w * t], Pc[[2]]\[Conjugate] * Exp[-I * w * t], Pc[[3]]\[Conjugate] * Exp[-I * w * t], 0}};

Grid[Hp]
```

$$\begin{matrix} 0 & 0 & 0 & \frac{e^{i t w}\,\text{hbar v}}{\sqrt{2}} \\ 0 & 0 & 0 & -\frac{e^{i t w}\,\text{hbar v}}{\sqrt{2}} \\ 0 & 0 & 0 & 0 \\ \frac{e^{-i t w}\,\text{hbar v}}{\sqrt{2}} & -\frac{e^{-i t w}\,\text{hbar v}}{\sqrt{2}} & 0 & 0 \end{matrix}$$

```
H = (Ho + Hp);

Grid[H]
```

$$\begin{matrix} \text{hbar}\,(-\text{Oe} + \text{wo}) & 0 & 0 & \frac{e^{i t w}\,\text{hbar v}}{\sqrt{2}} \\ 0 & \text{hbar}\,(\text{Oe} + \text{wo}) & 0 & -\frac{e^{i t w}\,\text{hbar v}}{\sqrt{2}} \\ 0 & 0 & \text{hbar wo} & 0 \\ \frac{e^{-i t w}\,\text{hbar v}}{\sqrt{2}} & -\frac{e^{-i t w}\,\text{hbar v}}{\sqrt{2}} & 0 & 0 \end{matrix}$$

```
(* Calculate -(i/hbar)[H,s], which is equal to ds/dt - master equation *)

sd = TrigExpand[FullSimplify[-(I / hbar) * (H.s - s.H)]] ;

(* Go to interaction-type picture -
 the For loop is because changing pictures also changes the derivative *)

pd = FullSimplify[
  sd /. sgp → pgp * Exp[I * w * t] /. spg → ppg * Exp[-I * w * t] /. sgm → pgm * Exp[I * w * t] /.
     smg → pmg * Exp[-I * w * t] /. sgo → pgo * Exp[I * w * t] /. sog → pog * Exp[-I * w * t]];
For[i = 1, i ≤ 3, i++, pd[[i]][[4]] = pd[[i]][[4]] / Exp[I * w * t] + I * w * p[[i]][[4]];
 pd[[4]][[i]] = pd[[4]][[i]] / Exp[-I * w * t] + -I * w * p[[4]][[i]]]

(* Add spontaneous emission *)

pdfull = pd + G * {{-spp, -smp, -sop, -pgp / 2}, {-spm, -smm, -som, -pgm / 2},
     {-spo, -smo, -soo, -pgo / 2}, {-ppg / 2, -pmg / 2, -pog / 2, spp + smm + soo}};

(* Now reduce the number of variables, because p is Hermitian (spm=smp conj) *)
```

```
pdsubs = pdfull /. spm → smpa - I * smpb /. spo → sopa - I * sopb /. smo → soma - I * somb /.
      ppg → pgpa - I * pgpb /. pmg → pgma - I * pgmb /. pog → pgoa - I * pgob;

(* The next step is to set this all to zero,
subject to the constraint that spp+smm+soo+sgg = 1,
to find the steady state solution.  To help mathematica do the solution,
we're going to independently set the real and the imaginary parts to zero *)

(* Now write coeffs as real and imaginary parts *)

pdri =
  Simplify[pdsubs /. pgp → pgpa + I * pgpb /. pgm → pgma + I * pgmb /. pgo → pgoa + I * pgob /.
     smp → smpa + I * smpb /. sop → sopa + I * sopb /. som → soma + I * somb];

pdr = ComplexExpand[Re[pdri]];

pdi = ComplexExpand[Im[pdri]];

(* We only need the upper half because the matrices are Hermitian.  Also,
the diagonal is real.  And the lower,
right-hand equation isn't linearly independent. *)

termlistr =
  DeleteCases[Flatten[pdr * {{1, 1, 1, 1}, {0, 1, 1, 1}, {0, 0, 1, 1}, {0, 0, 0, 0}}], 0];

termlisti =
  DeleteCases[Flatten[pdi * {{0, 1, 1, 1}, {0, 0, 1, 1}, {0, 0, 0, 1}, {0, 0, 0, 0}}], 0];

termlist = FullSimplify[Join[termlistr, termlisti, {spp + smm + soo + sgg - 1}]];

sdsol = Solve[termlist == 0, varlist];


(*   OK, now we have the solution, we just need to calculate the intensities. *)

(* For light polarized in the x direction  *)

Ip = FullSimplify[ReplaceAll[(spp + smm + 2 * smpa + 2 * soo), sdsol] /. w → wo + dw]
```

$$\left\{\left(32\,Oe^2\,v^2\,\left(G^2+4\,Oe^2+2\,v^2\right)\right)\Big/\left(16\,dw^4\,\left(G^2+4\,Oe^2\right)+8\,dw^2\,\left(G^4-16\,Oe^4+6\,\left(G^2+4\,Oe^2\right)\,v^2+2\,v^4\right)+\left(G^2+4\,Oe^2+2\,v^2\right)\,\left(\left(G^2+4\,Oe^2\right)^2+2\,\left(5\,G^2+4\,Oe^2\right)\,v^2+16\,v^4\right)\right)\right\}$$

```
Ip /. Oe → 0
```

{0}

```
IplowS = FullSimplify[Simplify[ComplexExpand[Series[Ip, {v, 0, 2}]]]]
```

$$\left\{\frac{32\,Oe^2\,v^2}{16\,dw^4+8\,dw^2\,\left(G^2-4\,Oe^2\right)+\left(G^2+4\,Oe^2\right)^2}+O[v]^3\right\}$$

```
Iperp = FullSimplify[ReplaceAll[(spp + smm - 2 * smpa + 2 * soo), sdsol] /. w → wo + dw]
```

$$\left\{\left(8 v^2 \left(4 dw^2 \left(G^2+4 Oe^2\right)+\left(G^2+2 v^2\right)\left(G^2+4 Oe^2+2 v^2\right)\right)\right) \Big/ \left(16 dw^4 \left(G^2+4 Oe^2\right)+8 dw^2 \left(G^4-16 Oe^4+6\left(G^2+4 Oe^2\right) v^2+2 v^4\right)+\left(G^2+4 Oe^2+2 v^2\right)\left(\left(G^2+4 Oe^2\right)^2+2\left(5 G^2+4 Oe^2\right) v^2+16 v^4\right)\right)\right\}$$

```
IperplowS = FullSimplify[Series[Iperp /. Oe → 0, {v, 0, 2}]]
```

$$\left\{\frac{8 v^2}{4 dw^2+G^2}+O[v]^3\right\}$$

```
IplowS / IperplowS
```

$$\left\{\frac{4\left(4 dw^2+G^2\right) Oe^2}{16 dw^4+8 dw^2\left(G^2-4 Oe^2\right)+\left(G^2+4 Oe^2\right)^2}+O[v]^1\right\}$$

```
(* Now lets do the same thing for circularly polarized light *)

(* For light that is circularly polarized,
we use {I,1,0}/Sqrt[2] or {I,-1,0}/Sqrt[2] *)

Pxdk = {I, 1, 0} / Sqrt[2]
```

$$\left\{\frac{i}{\sqrt{2}}, \frac{1}{\sqrt{2}}, 0\right\}$$

```
(* Now we translate this into the atom coordinates *)

Pxyz = {Pxdk[[1]], Pxdk[[2]] * Cos[theta] - Pxdk[[3]] * Sin[theta],
  Pxdk[[3]] * Cos[theta] + Pxdk[[2]] * Sin[theta]}
```

$$\left\{\frac{i}{\sqrt{2}}, \frac{\mathrm{Cos}[theta]}{\sqrt{2}}, \frac{\mathrm{Sin}[theta]}{\sqrt{2}}\right\}$$

```
(* We now write this in terms of circular polarizations *)

Pc = {(Pxyz[[1]] + I * Pxyz[[2]]) / Sqrt[2],
  - (Pxyz[[1]] - I * Pxyz[[2]]) / Sqrt[2], Pxyz[[3]]}
```

$$\left\{\frac{\frac{i}{\sqrt{2}}+\frac{i\,\mathrm{Cos}[theta]}{\sqrt{2}}}{\sqrt{2}}, \frac{-\frac{i}{\sqrt{2}}+\frac{i\,\mathrm{Cos}[theta]}{\sqrt{2}}}{\sqrt{2}}, \frac{\mathrm{Sin}[theta]}{\sqrt{2}}\right\}$$

```
(* If v is proportional to the light amplitude and the dipole moment,
and the angular frequency of the light is w,
the perterbation on the Hamiltonian caused by the light is *)
```

```
Hp = hbar * v * {{0, 0, 0, Pc[[1]] * Exp[I * w * t]},
    {0, 0, 0, Pc[[2]] * Exp[I * w * t]}, {0, 0, 0, Pc[[3]] * Exp[I * w * t]},
    {Pc[[1]]\[Conjugate] * Exp[-I * w * t], Pc[[2]]\[Conjugate] * Exp[-I * w * t], Pc[[3]]\[Conjugate] * Exp[-I * w * t], 0}};

Grid[Hp]
```

$$\begin{array}{cccc}
0 & 0 & 0 & \frac{e^{i t w}\, hbar\, v \left(\frac{i}{\sqrt{2}}+\frac{i \, Cos[theta]}{\sqrt{2}}\right)}{\sqrt{2}} \\
0 & 0 & 0 & \frac{e^{i t w}\, hbar\, v \left(-\frac{i}{\sqrt{2}}+\frac{i \, Cos[theta]}{\sqrt{2}}\right)}{\sqrt{2}} \\
0 & 0 & 0 & \frac{e^{i t w}\, hbar\, v\, Sin[theta]}{\sqrt{2}} \\
\frac{1}{\sqrt{2}} e^{-i t w}\, hbar\, v \left(-\frac{i}{\sqrt{2}} - \frac{1}{\sqrt{2}} i\, Conjugate[Cos[theta]]\right) & \frac{1}{\sqrt{2}} e^{-i t w}\, hbar\, v \left(\frac{i}{\sqrt{2}} - \frac{1}{\sqrt{2}} i\, Conjugate[Cos[theta]]\right) & \frac{1}{\sqrt{2}} e^{-i t w}\, hbar\, v\, Conjugate[Sin[theta]] & 0
\end{array}$$

```
Grid[FullSimplify[Hp]]
```

$$\begin{array}{cccc}
0 & 0 & 0 & \frac{1}{2} i\, e^{i t w}\, hbar\, v\, (1 + Cos[theta]) \\
0 & 0 & 0 & \frac{1}{2} i\, e^{i t w}\, hbar\, v\, (-1 + Cos[theta]) \\
0 & 0 & 0 & \frac{e^{i t w}\, hbar\, v\, Sin[theta]}{\sqrt{2}} \\
-\frac{1}{2} i\, e^{-i t w}\, hbar\, v\, (1 + Cos[theta]) & i\, e^{-i t w}\, hbar\, v\, Sin\left[\frac{theta}{2}\right]^2 & \frac{e^{-i t w}\, hbar\, v\, Sin[theta]}{\sqrt{2}} & 0
\end{array}$$

```
H = (Ho + Hp);
```

```
Grid[H]
```

hbar (-Oe + wo)	0	0	$\frac{e^{i t w} \text{hbar } v \left(\frac{i}{\sqrt{2}}+\frac{i \text{Cos[theta]}}{\sqrt{2}}\right)}{\sqrt{2}}$
0	hbar (Oe + wo)	0	$\frac{e^{i t w} \text{hbar } v \left(-\frac{i}{\sqrt{2}}+\frac{i \text{Cos[theta]}}{\sqrt{2}}\right)}{\sqrt{2}}$
0	0	hbar wo	$\frac{e^{i t w} \text{hbar v Sin[theta]}}{\sqrt{2}}$
$\frac{1}{\sqrt{2}} e^{-i t w} \text{hbar } v \left(-\frac{i}{\sqrt{2}} - \frac{1}{\sqrt{2}} i \text{ Conjugate[Cos[theta]]}\right)$	$\frac{1}{\sqrt{2}} e^{-i t w} \text{hbar } v \left(\frac{i}{\sqrt{2}} - \frac{1}{\sqrt{2}} i \text{ Conjugate[Cos[theta]]}\right)$	$\frac{1}{\sqrt{2}} e^{-i t w} \text{hbar v Conjugate[Sin[theta]]}$	0

```
(* Calculate -(i/hbar)[H,s], which is equal to ds/dt - master equation *)

sd = TrigExpand[FullSimplify[-(I / hbar) * (H.s - s.H)]] ;

(* Go to interaction-type picture -
 the For loop is because changing pictures also changes the derivative *)

pd = FullSimplify[
   sd /. sgp → pgp * Exp[I * w * t] /. spg → ppg * Exp[-I * w * t] /. sgm → pgm * Exp[I * w * t] /.
      smg → pmg * Exp[-I * w * t] /. sgo → pgo * Exp[I * w * t] /. sog → pog * Exp[-I * w * t]];
For[i = 1, i ≤ 3, i++, pd[[i]][[4]] = pd[[i]][[4]] / Exp[I * w * t] + I * w * p[[i]][[4]];
 pd[[4]][[i]] = pd[[4]][[i]] / Exp[-I * w * t] + -I * w * p[[4]][[i]]]

(* Add spontaneous emission *)

pdfull = pd + G * {{-spp, -smp, -sop, -pgp / 2}, {-spm, -smm, -som, -pgm / 2},
      {-spo, -smo, -soo, -pgo / 2}, {-ppg / 2, -pmg / 2, -pog / 2, spp + smm + soo}};

(* Now reduce the number of variables, because p is Hermitian (spm=smp conj) *)

pdsubs = pdfull /. spm → smpa - I * smpb /. spo → sopa - I * sopb /. smo → soma - I * somb /.
      ppg → pgpa - I * pgpb /. pmg → pgma - I * pgmb /. pog → pgoa - I * pgob;

(* The next step is to set this all to zero,
subject to the constraint that spp+smm+soo+sgg = 1,
to find the steady state solution.  To help mathematica do the solution,
we're going to independently set the real and the imaginary parts to zero *)

(* Now write coeffs as real and imaginary parts *)

pdri =
   Simplify[pdsubs /. pgp → pgpa + I * pgpb /. pgm → pgma + I * pgmb /. pgo → pgoa + I * pgob /.
       smp → smpa + I * smpb /. sop → sopa + I * sopb /. som → soma + I * somb];

pdr = ComplexExpand[Re[pdri]];
```

```
pdi = ComplexExpand[Im[pdri]];

(* We only need the upper half because the matrices are Hermitian.  Also,
the diagonal is real.  And the lower,
right-hand equation isn't linearly independent. *)

termlistr =
  DeleteCases[Flatten[pdr * {{1, 1, 1, 1}, {0, 1, 1, 1}, {0, 0, 1, 1}, {0, 0, 0, 0}}], 0];

termlisti =
  DeleteCases[Flatten[pdi * {{0, 1, 1, 1}, {0, 0, 1, 1}, {0, 0, 0, 1}, {0, 0, 0, 0}}], 0];

termlist = FullSimplify[Join[termlistr, termlisti, {spp + smm + soo + sgg - 1}]];

sdsol = Solve[termlist == 0, varlist];

Irha = Simplify[ReplaceAll[(spp + smm + 2 * smpa), sdsol] /. w → wo + dw];

IrhalowS = Simplify[Simplify[ComplexExpand[Series[Irha, {v, 0, 2}]]]]
```

$$\left\{\frac{2\left(4\,\mathrm{dw}^2+\mathrm{G}^2+8\,\mathrm{Oe}^2-16\,\mathrm{dw}\,\mathrm{Oe}\,\mathrm{Cos}[\mathrm{theta}]+\left(4\,\mathrm{dw}^2+\mathrm{G}^2\right)\mathrm{Cos}[2\,\mathrm{theta}]\right)\mathrm{v}^2}{16\,\mathrm{dw}^4+8\,\mathrm{dw}^2\left(\mathrm{G}^2-4\,\mathrm{Oe}^2\right)+\left(\mathrm{G}^2+4\,\mathrm{Oe}^2\right)^2}+\mathrm{O}[\mathrm{v}]^3\right\}$$

```
IrhaoverIp = Simplify[IrhalowS / IplowS]
```

$$\left\{\frac{4\,\mathrm{dw}^2+\mathrm{G}^2+8\,\mathrm{Oe}^2-16\,\mathrm{dw}\,\mathrm{Oe}\,\mathrm{Cos}[\mathrm{theta}]+\left(4\,\mathrm{dw}^2+\mathrm{G}^2\right)\mathrm{Cos}[2\,\mathrm{theta}]}{16\,\mathrm{Oe}^2}+\mathrm{O}[\mathrm{v}]^1\right\}$$

```
Simplify[(IrhaoverIp -
   ((dw^2 + (G / 2) ^2 ) * Cos[theta]^2 - 2 * dw * Oe * Cos[theta] + Oe^2) / (2 * Oe^2))]
```

$$\{\mathrm{O}[\mathrm{v}]^1\}$$

```
Irhb = Simplify[ReplaceAll[2 * soo, sdsol] /. w → wo + dw];

IrhblowS = Simplify[Simplify[ComplexExpand[Series[Irhb, {v, 0, 2}]]]]
```

$$\left\{\frac{4\,\mathrm{Sin}[\mathrm{theta}]^2\,\mathrm{v}^2}{4\,\mathrm{dw}^2+\mathrm{G}^2}+\mathrm{O}[\mathrm{v}]^3\right\}$$

```
IrhboverIperp = Simplify[Irhb / IperplowS]
```

$$\left\{\frac{\mathrm{Sin}[\mathrm{theta}]^2}{2}+\mathrm{O}[\mathrm{v}]^1\right\}$$

```
Simplify[(IrhboverIperp - Sin[theta]^2 / 2)]
```

$$\{\mathrm{O}[\mathrm{v}]^1\}$$

B Mathematica code for the steady state, linear polarization calculation

```
Quit[]

xrp = Axp * Exp[I * (wo + dw + wl) * t]

Axp ⅇ^(ⅈ t (dw+wl+wo))

axp = Simplify[Solve[
   D[xrp, {t, 2}] + wo^2 * xrp + G * D[xrp, t] == (Fo / 2) * Exp[I * (wo + dw + wl) * t], {Axp}]]

{{Axp → - Fo / (2 (dw^2 - ⅈ G (wl + wo) + wl (wl + 2 wo) + dw (- ⅈ G + 2 (wl + wo))))}}

xrm = Axm * Exp[I * (wo + dw - wl) * t]

Axm ⅇ^(ⅈ t (dw-wl+wo))

axm = Simplify[Solve[
   D[xrm, {t, 2}] + wo^2 * xrm + G * D[xrm, t] == (Fo / 2) * Exp[I * (wo + dw - wl) * t], {Axm}]]

{{Axm → - Fo / (2 (dw^2 + wl (wl - 2 wo) + ⅈ G (wl - wo) + dw (- ⅈ G - 2 wl + 2 wo)))}}

yrp = Ayp * Exp[I * (wo + dw + wl) * t]

Ayp ⅇ^(ⅈ t (dw+wl+wo))

ayp = Simplify[Solve[D[yrp, {t, 2}] + wo^2 * yrp + G * D[yrp, t] ==
     - I (Fo / 2) * Exp[I * (wo + dw + wl) * t], {Ayp}]]

{{Ayp → (ⅈ Fo) / (2 (dw^2 - ⅈ G (wl + wo) + wl (wl + 2 wo) + dw (- ⅈ G + 2 (wl + wo))))}}

yrm = Aym * Exp[I * (wo + dw - wl) * t]

Aym ⅇ^(ⅈ t (dw-wl+wo))

aym = Simplify[Solve[D[yrm, {t, 2}] + wo^2 * yrm + G * D[yrm, t] ==
     I (Fo / 2) * Exp[I * (wo + dw - wl) * t], {Aym}]]

{{Aym → - (ⅈ Fo) / (2 (dw^2 + wl (wl - 2 wo) + ⅈ G (wl - wo) + dw (- ⅈ G - 2 wl + 2 wo)))}}

yyrp = ReplaceAll[yrp, ayp]

{(ⅈ ⅇ^(ⅈ t (dw+wl+wo)) Fo) / (2 (dw^2 - ⅈ G (wl + wo) + wl (wl + 2 wo) + dw (- ⅈ G + 2 (wl + wo))))}

yyrm = ReplaceAll[yrm, aym]

{- (ⅈ ⅇ^(ⅈ t (dw-wl+wo)) Fo) / (2 (dw^2 + wl (wl - 2 wo) + ⅈ G (wl - wo) + dw (- ⅈ G - 2 wl + 2 wo)))}

xxrp = ReplaceAll[xrp, axp]

{- (ⅇ^(ⅈ t (dw+wl+wo)) Fo) / (2 (dw^2 - ⅈ G (wl + wo) + wl (wl + 2 wo) + dw (- ⅈ G + 2 (wl + wo))))}
```

```
xxrm = ReplaceAll[xrm, axm]
```

$$\left\{-\left(e^{i\,t\,(dw-wl+wo)}\,Fo\right)\Big/\left(2\left(dw^2+wl\,(wl-2\,wo)+i\,G\,(wl-wo)+dw\,(-i\,G-2\,wl+2\,wo)\right)\right)\right\}$$

```
y = Simplify[((yyrp + yyrm) * Cos[wl * t] - (xxrp + xxrm) * Sin[wl * t])]
```

$$\left\{\frac{1}{2}\,Fo\left(-i\left(e^{i\,t\,(dw-wl+wo)}\Big/\left(dw^2+wl\,(wl-2\,wo)+i\,G\,(wl-wo)+dw\,(-i\,G-2\,wl+2\,wo)\right)-e^{i\,t\,(dw+wl+wo)}\Big/\left(dw^2-i\,G\,(wl+wo)+wl\,(wl+2\,wo)+dw\,(-i\,G+2\,(wl+wo))\right)\right)Cos[t\,wl]+\left(e^{i\,t\,(dw-wl+wo)}\Big/\left(dw^2+wl\,(wl-2\,wo)+i\,G\,(wl-wo)+dw\,(-i\,G-2\,wl+2\,wo)\right)+e^{i\,t\,(dw+wl+wo)}\Big/\left(dw^2-i\,G\,(wl+wo)+wl\,(wl+2\,wo)+dw\,(-i\,G+2\,(wl+wo))\right)\right)Sin[t\,wl]\right)\right\}$$

```
ystary = Simplify[ComplexExpand[y * Conjugate[y]]];

ystarysimp = Series[ystary /. wo → 1 / q, {q, 0, 2}]
```

$$\left\{\frac{4\,Fo^2\,wl^2\,q^2}{\left(4\,dw^2+G^2-8\,dw\,wl+4\,wl^2\right)\left(4\,dw^2+G^2+8\,dw\,wl+4\,wl^2\right)}+O[q]^3\right\}$$

```
Simplify[ystarysimp /
  ((wl^2 * Fo^2 * q^2 / 4) / (dw^4 + 2 * dw^2 * ((G / 2)^2 - wl^2) + ((G / 2)^2 + wl^2)^2))]
```

$$\left\{1+O[q]^1\right\}$$

C Mathematica code for the steady state, circular polarization calculation

```
Quit[]
```

```
xrp = Axp * Exp[I * (wo + dw + wl) * t]
```

$$\text{Axp}\, e^{i\, t\, (\text{dw}+\text{wl}+\text{wo})}$$

```
axp = Simplify[Solve[D[xrp, {t, 2}] + wo^2 * xrp + G * D[xrp, t] ==
    (Fo / (2 * Sqrt[2])) * (1 + Cos[theta]) * Exp[I * (wo + dw + wl) * t], {Axp}]]
```

$$\left\{\left\{\text{Axp} \to -\frac{\text{Fo}\,(1+\text{Cos}[\text{theta}])}{2\sqrt{2}\,\left(\text{dw}^2 - i\,G\,(\text{wl}+\text{wo}) + \text{wl}\,(\text{wl}+2\,\text{wo}) + \text{dw}\,(-i\,G + 2\,(\text{wl}+\text{wo}))\right)}\right\}\right\}$$

```
xrm = Axm * Exp[I * (wo + dw - wl) * t]
```

$$\text{Axm}\, e^{i\, t\, (\text{dw}-\text{wl}+\text{wo})}$$

```
axm = Simplify[Solve[D[xrm, {t, 2}] + wo^2 * xrm + G * D[xrm, t] ==
    (Fo / (2 * Sqrt[2])) * (1 - Cos[theta]) * Exp[I * (wo + dw - wl) * t], Axm]]
```

$$\left\{\left\{\text{Axm} \to \frac{\text{Fo}\,(-1+\text{Cos}[\text{theta}])}{2\sqrt{2}\,\left(\text{dw}^2 + \text{wl}\,(\text{wl}-2\,\text{wo}) + i\,G\,(\text{wl}-\text{wo}) + \text{dw}\,(-i\,G - 2\,\text{wl} + 2\,\text{wo})\right)}\right\}\right\}$$

```
yrp = Ayp * Exp[I * (wo + dw + wl) * t]
```

$$\text{Ayp}\, e^{i\, t\, (\text{dw}+\text{wl}+\text{wo})}$$

```
ayp = Simplify[Solve[D[yrp, {t, 2}] + wo^2 * yrp + G * D[yrp, t] ==
    -I * (Fo / (2 * Sqrt[2])) * (1 + Cos[theta]) * Exp[I * (wo + dw + wl) * t], {Ayp}]]
```

$$\left\{\left\{\text{Ayp} \to (i\,\text{Fo}\,(1+\text{Cos}[\text{theta}]))\Big/\left(2\sqrt{2}\,\left(\text{dw}^2 - i\,G\,(\text{wl}+\text{wo}) + \text{wl}\,(\text{wl}+2\,\text{wo}) + \text{dw}\,(-i\,G + 2\,(\text{wl}+\text{wo}))\right)\right)\right\}\right\}$$

```
yrm = Aym * Exp[I * (wo + dw - wl) * t]
```

$$\text{Aym}\, e^{i\, t\, (\text{dw}-\text{wl}+\text{wo})}$$

```
aym = Simplify[Solve[D[yrm, {t, 2}] + wo^2 * yrm + G * D[yrm, t] ==
    I * (Fo / (2 * Sqrt[2])) * (1 - Cos[theta]) * Exp[I * (wo + dw - wl) * t], {Aym}]]
```

$$\left\{\left\{\text{Aym} \to (i\,\text{Fo}\,(-1+\text{Cos}[\text{theta}]))\Big/\left(2\sqrt{2}\,\left(\text{dw}^2 + \text{wl}\,(\text{wl}-2\,\text{wo}) + i\,G\,(\text{wl}-\text{wo}) + \text{dw}\,(-i\,G - 2\,\text{wl} + 2\,\text{wo})\right)\right)\right\}\right\}$$

```
yyrp = ReplaceAll[yrp, ayp][[1]]
```

$$\left(i\, e^{i\, t\, (\text{dw}+\text{wl}+\text{wo})}\,\text{Fo}\,(1+\text{Cos}[\text{theta}])\right)\Big/\left(2\sqrt{2}\,\left(\text{dw}^2 - i\,G\,(\text{wl}+\text{wo}) + \text{wl}\,(\text{wl}+2\,\text{wo}) + \text{dw}\,(-i\,G + 2\,(\text{wl}+\text{wo}))\right)\right)$$

```
yyrm = ReplaceAll[yrm, aym][[1]]
```

$$\frac{i\, e^{i\, t\, (dw-wl+wo)}\, Fo\, (-1 + Cos[theta])}{2\sqrt{2}\, \left(dw^2 + wl\, (wl - 2\, wo) + i\, G\, (wl - wo) + dw\, (-i\, G - 2\, wl + 2\, wo)\right)}$$

```
xxrp = ReplaceAll[xrp, axp][[1]]
```

$$-\left(e^{i\, t\, (dw+wl+wo)}\, Fo\, (1 + Cos[theta])\right) \Big/ \left(2\sqrt{2}\, \left(dw^2 - i\, G\, (wl + wo) + wl\, (wl + 2\, wo) + dw\, (-i\, G + 2\, (wl + wo))\right)\right)$$

```
xxrm = ReplaceAll[xrm, axm][[1]]
```

$$\frac{e^{i\, t\, (dw-wl+wo)}\, Fo\, (-1 + Cos[theta])}{2\sqrt{2}\, \left(dw^2 + wl\, (wl - 2\, wo) + i\, G\, (wl - wo) + dw\, (-i\, G - 2\, wl + 2\, wo)\right)}$$

```
yp = Simplify[yyrp * Cos[wl * t] - xxrp * Sin[wl * t]]
```

$$\left(i\, e^{i\, t\, (dw+wl+wo)}\, Fo\, (1 + Cos[theta])\, (Cos[t\, wl] - i\, Sin[t\, wl])\right) \Big/ \left(2\sqrt{2}\, \left(dw^2 - i\, G\, (wl + wo) + wl\, (wl + 2\, wo) + dw\, (-i\, G + 2\, (wl + wo))\right)\right)$$

```
ym = Simplify[yyrm * Cos[wl * t] - xxrm * Sin[wl * t]]
```

$$\frac{i\, e^{i\, t\, (dw-wl+wo)}\, Fo\, (-1 + Cos[theta])\, (Cos[t\, wl] + i\, Sin[t\, wl])}{2\sqrt{2}\, \left(dw^2 + wl\, (wl - 2\, wo) + i\, G\, (wl - wo) + dw\, (-i\, G - 2\, wl + 2\, wo)\right)}$$

```
y = yp + ym
```

$$\left(i\, e^{i\, t\, (dw+wl+wo)}\, Fo\, (1 + Cos[theta])\, (Cos[t\, wl] - i\, Sin[t\, wl])\right) \Big/ \left(2\sqrt{2}\, \left(dw^2 - i\, G\, (wl + wo) + wl\, (wl + 2\, wo) + dw\, (-i\, G + 2\, (wl + wo))\right)\right) + \left(i\, e^{i\, t\, (dw-wl+wo)}\, Fo\, (-1 + Cos[theta])\, (Cos[t\, wl] + i\, Sin[t\, wl])\right) \Big/ \left(2\sqrt{2}\, \left(dw^2 + wl\, (wl - 2\, wo) + i\, G\, (wl - wo) + dw\, (-i\, G - 2\, wl + 2\, wo)\right)\right)$$

```
ystary = Simplify[ComplexExpand[y * Conjugate[y]]];
```

```
ystarysimp = Series[ystary /. wo → 1 / q, {q, 0, 2}]
```

$$\left(Fo^2\, \left(4\, dw^2 + G^2 + 8\, wl^2 - 16\, dw\, wl\, Cos[theta] + 4\, dw^2\, Cos[2\, theta] + G^2\, Cos[2\, theta]\right)\, q^2\right) \Big/ \left(4\, \left(4\, dw^2 + G^2 - 8\, dw\, wl + 4\, wl^2\right)\, \left(4\, dw^2 + G^2 + 8\, dw\, wl + 4\, wl^2\right)\right) + O[q]^3$$

```
Ip = (wl^2 * Fo^2 * q^2 / 4) / (dw^4 + 2 * dw^2 * ((G / 2)^2 - wl^2) + ((G / 2)^2 + wl^2)^2)
```

$$\frac{Fo^2\, q^2\, wl^2}{4\, \left(dw^4 + 2\, dw^2\, \left(\frac{G^2}{4} - wl^2\right) + \left(\frac{G^2}{4} + wl^2\right)^2\right)}$$

```
FullSimplify[ystarysimp /
  ((Ip / (2 * wl^2)) * ((dw^2 + (G / 2)^2) * Cos[theta]^2 - 2 * dw * wl * Cos[theta] + wl^2))]
```

$$1 + O[q]^1$$
